# Chicken Embryo Extract Remodeling of Extracellular Matrix Sustains Self-Renewal and Differentiation for Scaffold-Free Cell Sheet Formation

**DOI:** 10.34133/bmr.0332

**Published:** 2026-03-03

**Authors:** Jeong-Eun Lee, Jin-Ryong Park, Yoo-Kyung Kang, Kwan-Seob Shim, Jeong-Tae Do

**Affiliations:** ^1^ School of Advanced Biotechnology, Konkuk University, Seoul, Republic of Korea.; ^2^ Food Processing Research Group, Korea Food Research Institute, Wanju, Republic of Korea.; ^3^ Department of Animal Biotechnology, Jeonbuk National University, Jeonju, Republic of Korea.; ^4^ Department of Agricultural Convergence Technology, Jeonbuk National University, Jeonju, Republic of Korea.

## Abstract

Fetal bovine serum (FBS) is commonly used in cell culture but can make up to 60% of total production costs, limiting the scalability of cultured meat (CM). Here, we investigated chicken embryo extract (CEE) as a functional and cost-effective substitute for FBS in culturing porcine muscle satellite cells and generating scaffold-free CM constructs. The 20% CEE + 5% horse serum (HS) medium enhanced myogenic cell growth and development while maintaining paired box 7 expression and up-regulating Myogenin, supporting the coexistence of self-renewing and differentiating states. Oxygen consumption and gene expression analyses revealed reduced oxidative metabolism alongside activation of self-renewal pathways. Transcriptomic analysis showed a specific increase in growth-factor-related genes in 20% CEE + 5% HS group, including *CXCL12*, *TGFB3*, and *FGF1*. Furthermore, 20% CEE + 5% HS differentiation media promoted the extracellular matrix and stable cell sheet organization. Stacked CEE-derived sheets yielded CM constructs with hardness and chewiness levels comparable to those of conventional pork cuts, while maintaining similar springiness and cohesiveness. Our findings show that 20% CEE + 5% HS is a feasible and cost-effective alternative to FBS, allowing for dual cell fate regulation and facilitates structured CM.

## Introduction

The successful commercialization of cultured meat (CM) critically depends on overcoming the challenge of low-cost mass production [1]. Several technical issues must be resolved before this can be accomplished [2]. One crucial element is the culture medium, which constitutes a substantial portion of production costs and plays a vital role in influencing the metabolic pathways and physiological characteristics of cells. Therefore, for efficient production of CM, research and development of a cost-effective medium that can enhance cultivation efficiency are necessary [3].

Fetal bovine serum (FBS) is widely used in diverse cell culture systems, including the cultivation of muscle satellite cells (SCs), owing to its substantial reservoir of essential nutrients, growth factors, hormones, and proteins that are critical for facilitating cell growth and ensuring viability [4,5]. Nonetheless, producing FBS at the laboratory level is challenging because it is derived from the serum of calves from slaughtered pregnant cows [3]. In addition, FBS accounts for over 60% of the total culture medium cost [5]. Thus, the development of cost-effective serum alternatives remains a critical challenge for CM production [5]. However, developing a substitute with all the essential attributes of FBS has been a costly endeavor [4]. Moreover, the development of an affordable and easily producible culture medium that can efficiently enhance the proliferation, differentiation, and tissue engineering of muscle cells remains a substantial limitation.

Eggs contain all the growth factors necessary for embryonic development, enabling chicks to fully mature in 21 d without external nutritional input [6]. Chicken embryo extract (CEE) is recognized for its diverse growth factor content that promotes cell growth and development [6,7]. Therefore, CEE has been used as a supplement at 0.5% to 5% concentrations for the cultivation of fibroblasts, neural crest stem cells, muscle cells, and mesenchymal stem cells [6–9]. While the precise mechanisms of CEE are still unclear, recent evidence indicates that it contains nutritional factors and cytokines capable of promoting cell proliferation and differentiation [7]. However, there are still a few studies investigating the impact of using CEE as an alternative to FBS, particularly in the context of CM production. Since eggs offer a more accessible and cost-effective alternative in comparison to FBS or other synthetic supplements [10,11], CEE has been used as a useful additive in muscle cell culture.

Previous studies have used CEE mainly as a minor additive rather than as a primary component. In this study, we apply CEE as the main source for cultured porcine muscle SCs (PMSCs), demonstrating its effectiveness in both proliferation and differentiation compared with FBS-based conditions. We also developed an optimized in-house CEE production that showed higher efficacy. In addition, we evaluated the ability of the CEE-based culture system to support advanced tissue engineering, including myotube formation in cell sheets. Typically, tissue engineering techniques require a supply of extracellular matrix (ECM) components and a scaffold during the culture period [12,13]. Here, we established a method to generate thick patties by sequentially layering scaffold-free myotube cell sheets using a CEE-based culture system. Consequently, our study expanded the applicability of CEE in tissue engineering and CM production.

## Materials and Methods

### Isolation of the PMSCs

PMSCs were isolated from the biceps femoris muscle of 1-d-old crossbred piglets (Landrace × Yorkshire × Duroc), which were sourced from a local pig farm in Korea. Muscle tissues were minced and digested in Dulbecco’s Modified Eagle Medium/Nutrient Mixture F–12 (DMEM/F12, Gibco) with collagenase D (2 mg/ml; Roche), dispase II (1 U/ml; Roche), 0.25% trypsin-EDTA (Gibco) and 10% penicillin–streptomycin (PS; Gibco) for 1 h at 37 °C. The digested solutions were neutralized with 10% FBS and 1% penicillin–streptomycin–glutamine (PSG; Gibco) in DMEM/F12 (Gibco). The cells were then sequentially filtered through 100- and 70-μm cell strainers and centrifuged at 204×*g* for 5 min. Ammonium–chloride–potassium lysis buffer (Gibco) was added to the pellets. After centrifugation, the ammonium–chloride–potassium lysis buffer was removed, and the cells were suspended in proliferation media (PM) (DMEM/F12, 15% FBS, 1% PSG, and 5 ng/ml basic fibroblast growth factor (bFGF; Gibco). Purification of SCs was achieved using the preplating methods [14], and the presence of PMSCs was confirmed using SC surface markers with a BD FACSDiva 9.1 (BD Biosciences; Fig. S1).

### CEE

**Fig. 1.**
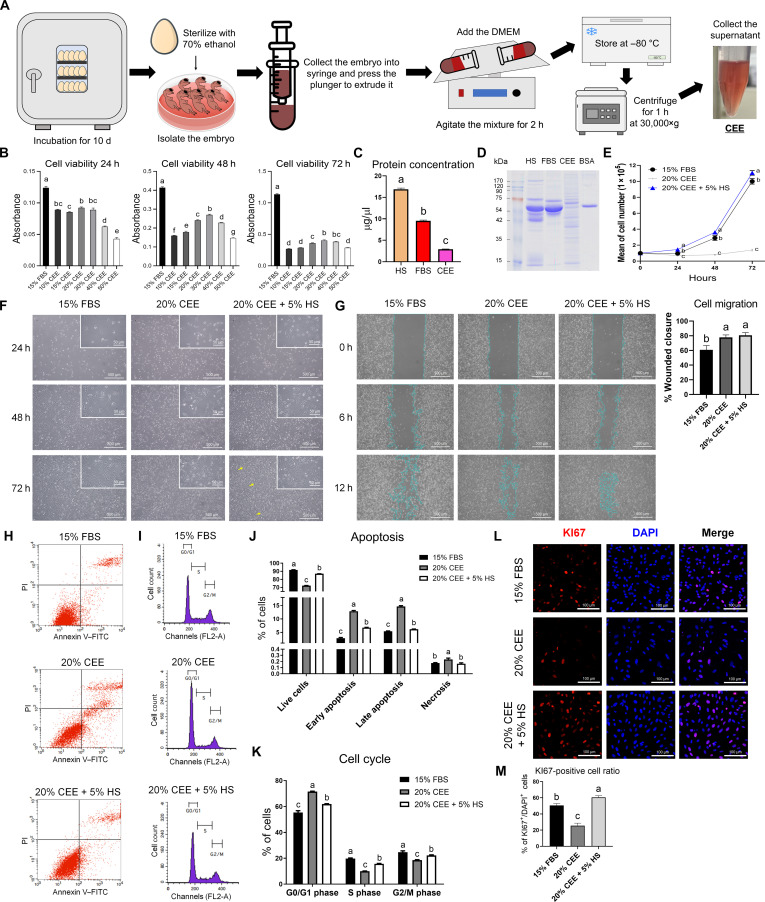
Systematic optimization of CEE-enriched media to enhance the proliferative potential of PMSCs. (A) Schematic diagram of CEE production. (B) CEE-induced alterations in PMSC viability in a time- and dose-dependent manner in 6 replicates. (C) Protein concentrations in equal amounts of FBS, HS, and CEE. (D) Electrophoretic pattern of serum proteins. BSA was used as a positive control. (E) Cell proliferation curve for 15% FBS, 20% CEE, and 20% CEE + 5% HS. (F) Cell morphology was observed using optical microscopy. Yellow arrowheads indicate regions showing myotube morphology. (G) Cell migration after 12 h under 20% CEE or 20% CEE + 5% HS. (H) Flow cytometry analysis of apoptosis using annexin V and PI staining. (I) The cell cycle of PMSCs was detected using PI staining. (J and K) Percentages of live, apoptotic, necrotic cells, and cell cycle distribution under different media. (L) Immunofluorescent staining results for KI67 (red) and DAPI (blue). (M) The percentage of KI67-positive cells was determined by counting the KI67-positive cells relative to the total number of DAPI-positive cells. All values are represented as means ± SE. ^a-g^Different letters indicate statistically significant differences (*P* < 0.01), and the shared letters (e.g., ^b^ and ^bc^) indicate no differences.

To prepare CEE, 10-d-old chicken embryos were used (Fig. 1A), as this stage coincides with active organogenesis and myogenesis, providing abundant soluble growth factors while reducing contamination from keratinized or fibrous tissues that develop in later stages. The eggs were sterilized with 70% ethanol and transferred to a clean bench. All embryos were immediately extracted from their eggshells. The embryos were washed with DMEM/F12 containing 10% PS. Subsequently, the embryos were collected using a syringe and homogenized. The extract was diluted with DMEM/F12 and mixed using an agitator for 2 h. The mixture was stored at −80 °C for 48 h and thawed at 37 °C. The thawed mixture was centrifuged at 500×*g* and 30,000×*g* for 1 h. The supernatant was collected and frozen at −80 °C. Approximately 4 to 5 ml of CEE were obtained from a single chicken embryo. Before use, the CEE was centrifuged at 1,000×*g* for 10 min and filtered through 0.22-μm filters.

### Cell culture for proliferation conditions

All culture dishes were coated with 0.15% gelatin before seeding the cells and were maintained in a humidified incubator at 37 °C with 5% CO_2_. PMSCs ranging from passages 1 to 4 were used for the experiments. For each analysis type, cells from the same passage were used to ensure consistency, and early passages were expanded to increase the total cell yield required for downstream assays. Based on the PM used in our previous study [14] on PMSCs, 15% FBS was established as a control. For CEE treatments, FBS was replaced with CEE at designated concentrations (10%, 15%, 20%, 30%, 40%, and 50%). After determining the baseline CEE concentration, 20% CEE and 30% CEE were supplemented with 1%, 3%, and 5% FBS and 1%, 3%, and 5% horse serum (HS) (Gibco), respectively. The optimal CEE concentration, determined by Cell Counting Kit-8 (CCK-8) assay and morphological evaluation, was used for subsequent experiments. Morphology images were acquired using an Olympus CKX41 microscope. The chosen culture conditions in proliferation stage were as follows: 15% FBS (DMEM/F12, 15% FBS, 1% PSG, and 5 ng/ml bFGF), 20% CEE (DMEM/F12, 20% CEE, 1% PSG, and 5 ng/ml bFGF), and 20% CEE + 5% HS (DMEM/F12, 20% CEE, 5% HS, 1% PSG, and 5 ng/ml bFGF). The culture medium was replaced every 24 h. For additional analyses of mRNA and protein expression under proliferation conditions, PMSCs were harvested at day 3, before reaching full confluency (~100%).

### Cell viability and proliferation assay

PMSCs were suspended in each medium and seeded in 96-well plates at a density of 5 × 10^3^ cells per well. Cell viability was assessed at 24, 48, and 72 h using the CCK-8 assay (Dojindo). CCK-8 solution was added to each well and incubated for 4 h in a 5% CO_2_ incubator at 37 °C. Absorbance was measured at 450 nm using a microplate reader (Thermo Fisher Scientific). For the proliferation assay, PMSCs were seeded in triplicate into 6-well plates at a density of 1 × 10^5^ cells per well. The cells were detached with 0.25% trypsin-EDTA, and cell counts were performed using a hemocytometer under an inverted microscope.

### Protein extraction and gel electrophoresis of serum samples

Equal amounts of FBS, HS, and CEE were lysed in 5× phosphate-buffered saline (PBS). After sonication for 5 s, the diluted sera were placed on ice for 50 s, and this cycle was repeated 6 times. Subsequently, the samples were centrifuged at 21,000×*g* for 30 min, and the supernatants were collected. The protein concentration was measured using a detergent-compatible (DC) protein assay kit (Bio-Rad). Protein (40 μg) was mixed with sample buffer (Bio-Rad) and heated at 95 °C for 5 min. The samples were then separated on 12% acrylamide gels and stained with Coomassie Brilliant Blue R-250 solution (Bio-Rad) at room temperature (RT) for 1 h. Next, the stained gel was incubated in 20% methanol and 10% glacial acetic acid in distilled water at RT overnight. Protein bands were captured using Image Master 2D Platinum (GE HealthCare).

### Wound healing assay

PMSCs were seeded in 6-well plates at a density of 1.5 × 10^5^ cells per well and cultured until 95% confluence. Mitomycin C (Sigma-Aldrich) was applied at 10 μg/ml for 2 h. The cells were scratched using 1,000-μl tips and washed with PBS. The cell-free area was assessed using the ImageJ software on the captured images. Wounded area results were normalized to the 0-h wounded area, and the percentage wound closure was calculated.

### Cell cycle and apoptosis analysis

After 48 h of culture, the cells were harvested and washed with PBS. For cell cycle analysis, the cells were fixed with cold 70% ethanol and kept at 4 °C for 5 min. After fixation, the cells were centrifuged at 850×*g* for 5 min and washed with cold PBS containing 1% bovine serum albumin (BSA). The cells were then resuspended in 100 μg/ml ribonuclease A (Thermo Fisher Scientific) and stained with 500 μg/ml propidium iodide (PI) solution (BioLegend). For the apoptosis analysis, a fluorescein isothiocyanate annexin V apoptosis detection kit was used, following the manufacturer’s instructions. The cells were subjected to a double-staining procedure using fluorescein isothiocyanate–annexin V and PI solution for 15 min at RT in the dark. Subsequently, the cells were suspended in annexin V binding buffer and stored on ice prior to analysis. Cell cycle and apoptosis were determined using a FACSCalibur flow cytometer (Becton) and BD CellQuest Pro software.

### Immunofluorescent staining

PMSCs were seeded at 1 × 10^5^ cells per well in confocal dishes. The cells were fixed with 4% paraformaldehyde for 20 min at 4 °C after 48 h of culture. For differentiation experiments, the PMSCs in differentiation medium (DM) were fixed with 4% paraformaldehyde for 20 min at 4 °C after 7 d of differentiation induction. Subsequently, the cells were rinsed with PBS and treated with a blocking solution (PBS containing 3% BSA and 0.3% Triton X-100) for 1 h at RT. Primary antibodies were diluted in blocking solution at the following ratios: anti-KI67 (1:100; GeneTex), anti-PAX7 (paired box 7) (1:20; Developmental Studies Hybridoma Bank [DSHB]), anti-MYOD1 (myogenic differentiation 1) (1:200; Proteintech), anti-Myogenin (monoclonal, 1:100; DSHB), and myosin heavy chain (MYHC) (1:20; DSHB). The primary antibodies were then incubated overnight at 4 °C, and the cells were subsequently incubated with secondary antibodies (Alexa Fluor 488 or 568, Eugene) for 2 h at RT. Cell nuclei were stained with 4′-6-diamidino-2-phenylindole (DAPI; Sigma-Aldrich). Fluorescence and Z-stack images were acquired using a super-resolution confocal laser scanning microscope (LSM 880, Carl Zeiss) and analyzed using ZEN imaging software (Carl Zeiss). The percentage of positive cells was assessed by investigators blinded to the experimental treatment group.

### Western blotting

The cell pellet was resuspended in 200 μl of radioimmunoprecipitation assay buffer (Biosesang) containing a protease inhibitor (Thermo Fisher Scientific) to extract total proteins. The concentration of the extracted protein was measured using a DC Protein Assay Kit (Bio-Rad). Equal amounts (20 μg/μl) of protein samples were combined with sample buffer (Bio-Rad) and heated at 95 °C for 5 min. The protein samples were separated on 12% acrylamide gel. The separated protein bands were transferred onto polyvinylidene fluoride membranes (Bio-Rad). The membranes were blocked using blocking buffer (Bio-Rad). Each membrane was incubated overnight at 4 °C with the following antibodies: PAX7 (1:50; DSHB), MYOD1 (1:1,000; Proteintech), Myogenin (1:50; DSHB), and glyceraldehyde 3-phosphate dehydrogenase (GAPDH) (1: 5,000, Invitrogen). The secondary antibodies were diluted at a dilution ratio twice that of each primary antibody and incubated with the membranes for 2 h at RT. Protein bands were visualized using SuperSignal chemiluminescent substrates (Thermo Fisher Scientific) and imaged using the iBright CL100 Imaging System (Thermo Fisher Scientific). All protein data were normalized against GAPDH.

### RT-qPCR

Total RNA was extracted using TRIzol reagent (15596026, Invitrogen) and precipitated with isopropanol. The total RNA concentration was quantified using a NanoDrop spectrophotometer (Thermo Fisher Scientific). Complementary DNA was synthesized from 1 μg of RNA using SuperScript III reverse transcriptase (Invitrogen), poly T oligonucleotides (Invitrogen), and 10 mM dNTP Mix (Invitrogen) according to the manufacturer’s instructions. Reverse transcription quantitative polymerase chain reaction (RT-qPCR) was performed using TOPreal qPCR 2× premix (Enzynomics) on a Roche LightCycler 5480 platform (Roche). The primer sequences used for RT-qPCR are shown in Table S1. The amplification cycle consisted of 40 cycles at the following temperatures: 95 °C for 10 s, 60 °C for 10 s, and 72 °C for 20 s. All relative mRNA expression data were normalized to GAPDH and calculated using the 2^−ΔΔCt^ method.

### Mitochondrial respiration analysis

Oxygen consumption rate (OCR) was assessed using an XFp analyzer (Seahorse Bioscience) following the manufacturer’s instructions. PMSCs (2 × 10^4^) were seeded Agilent Seahorse XFp well plates and cultured for 24 h using each medium. An hour before the analysis, the medium was replaced with XFp base medium supplemented with glucose (Agilent), sodium pyruvate (Agilent), and glutamine (Agilent). To inhibit mitochondrial respiration, oligomycin (1.5 μM), carbonyl cyanide *p*-trifluoromethoxyphenylhydrazone (2.0 μM), and rotenone/antimycin A (0.5 μM) were sequentially injected. OCR was normalized to total protein concentration using a DC Protein Assay Kit (Bio-Rad).

### Myogenic differentiation and analysis

PMSCs were seeded at a density of 1 × 10^5^ cells per well in 6-well plates and cultured to 90% to 100% confluence in their respective PM for 3 d. To induce differentiation, the PM was replaced with DM. High-glucose DMEM (Hyclone) was used as the basal medium for all DM in this study. Under condition 1, 5% HS and 1% PSG were used for DM. For conditions 2 and 3, 20% CEE, 5% HS, and 1% PSG were used. The DM was changed every 2 d. After 7 d of culture in the DM, cell pellets were collected. All subsequent analyses related to differentiation were performed using samples collected at this time point. Wet weight of each sample was calculated by subtracting the weight of the empty tube from the total mass. Protein extraction and quantification were performed as described above. All analyses were conducted in triplicate.

### Live/dead cell staining

The single-layer cell sheets were stained using the Live/Dead Cell Imaging Kit (Thermo Fisher Scientific) according to the manufacturer’s instructions. Live-green and dead-red reagents were combined to create a 2× stock solution, which was then added to the cell sheets for 15 min at RT, followed by observation using fluorescence microscopy.

### Preparation of stacked cell sheets using CEE

PMSCs were seeded at a density of 5 × 10^4^ cells per well (24 wells) or 1 × 10^5^ cells per well (6 wells) under condition 2 to generate the cell sheets. After 5 d of differentiation, the cell sheets were detached using a tip or forceps and transferred to 3.5-cm^2^ dishes and sequentially stacked with a 15-min incubation after each layer to promote adherence. The stacked 10-layer cell sheets were then incubated for 3 d.

### RNA-seq

To investigate gene expression changes, RNA sequencing (RNA-seq) was conducted based on 2 main comparisons: (a) 15% FBS versus 20% CEE + 5% HS PM group and (b) condition 1 versus condition 2 DM groups. Sequencing was performed by Macrogen (Seoul, Republic of Korea). RNA was extracted using an RNeasy Mini Kit (QIAGEN). To remove DNA contamination, RNA was treated with ribonuclease-free deoxyribonuclease I (QIAGEN). Purified RNA was evaluated using a NanoDrop 2000 spectrophotometer (Thermo Fisher Scientific). The TruSeq Stranded mRNA Library Prep Kit (Illumina Inc.) was used to construct the complementary DNA libraries. Total RNA was sequenced using the Illumina NovaSeq 6000 system (Macrogen). The quality of the raw RNA-seq data was evaluated using FastQC (version 0.11.7) software, and low-quality and adapter sequences were removed using Trimmomatic (version 0.38) software. All libraries were individually mapped to a genomic DNA reference (Sscrofa 11.1) using HISAT2 (version 2.1.0). The mapped reads were assembled using the String Tie software (version 2.1.3b). Based on these genes, the read count, fragments per kilobase of transcript per million mapped reads (FPKM), and transcripts per kilobase per million were determined.

### Bioinformatics analysis

Differentially expressed genes (DEGs) were identified on the basis of a fold change (FC) ≥ 2 and a *P* < 0.05. Heatmaps were generated using the heatmap.2 tool in R and clustered using the hclust tool. Gene ontology (GO) enrichment analysis was performed using DAVID web tools and visualized in R with ggplot2 (bar plot or bubble plot) using the R software. Scatter plots were generated using the base plot function in R software, with genes classified using FC ≥ 2, *P* < 0.05, and FPKM ≥ 2. DEGs were mapped to the Kyoto Encyclopedia of Genes and Genomes (KEGG) pathway database using the pathview package in R (FC ≥ 2).

### Histological analysis of stacked cell sheets

Hematoxylin and eosin (H&E) and Masson’s trichrome staining were performed to assess the structure. The stacked cell sheets were fixed in 4% paraformaldehyde for 24 h at 4 °C, embedded in paraffin, and sectioned into 5-μm slices. The blocks were then deparaffinized and hydrated with ethanol. The samples were stained with H&E and trichrome staining solutions. Images of the stained samples were captured using a microscopic imaging system.

### Texture profile analysis

To evaluate the texture profile, cell-sheet-based CM and commercial meats, such as defrosted shoulder butt (SB), refrigerated tenderloin (TL), and press ham (PH), were uniformly sized prior to texture measurements. Texture profile analysis (TPA) was performed using a texture analyzer (TA.XTPlus) with a spherical probe. The samples were compressed to 20% of their original height at a rate of 30 mm/min. The trigger force was set to 5 g. Texture parameters, such as hardness, springiness, chewiness, and cohesiveness, were calculated from the TPA curve.

### Diameter and thickness measurements of the layered cell sheets

The diameters and thicknesses of the raw and fried multilayered cell sheets were quantified using the ImageJ software based on top- and side-view images. Top-view images were used for diameter measurements, and 4 measurements were taken per sample. The thickness was assessed using side-view images by measuring 3 evenly spaced points for each sample. All measurements were performed under identical imaging conditions to ensure consistency of the results. Measurements were performed using 3 biological replicates.

### Statistical analysis

Statistical analyses of all data were performed using SAS software (version 9.4; SAS Institute Inc., Cary, USA). One-way analysis of variance (ANOVA) followed by Duncan’s multiple range test was used to compare the statistically significant differences within each group. Analysis of variance, followed by Student *t* test, was used to compare raw and fried layered cell sheets. All values are represented as means ± standard error (SE), and *P* < 0.01 and *P* < 0.05 were considered statistically significant.

## Results

### PMSC proliferation supported by 20% CEE + 5% HS

The quality of CEE can vary depending on differences in production methods and source materials [10]. Therefore, we optimized the methods for CEE extraction (Fig. 1A), focusing in particular on centrifugation speed. We found that 30,000×*g* was more suitable for PMSC culture, and this condition was subsequently applied in CEE preparation (Fig. S2A). We then observed the morphology of PMSCs according to the CEE concentration (10%, 15%, 20%, 30%, 40%, and 50%) (Fig. S2B). The short spindle-shaped characteristic of PMSCs was maintained for 72 h in the 15% FBS group, whereas those cultured in CEE medium exhibited an elongated phenotype. Among the CEE treatments, 30% CEE exhibited the highest cell viability (*P* < 0.01) at 48 and 72 h (Fig. 1B). However, cell viability remained lower than that observed in the 15% FBS group. Nevertheless, our in-house prepared CEE exhibited markedly higher cell proliferation and viability than the commercial CEE (*P* < 0.01), highlighting the importance of optimized CEE preparation methods for effective PMSC culture (Fig. S2C and D).

To investigate the cause of the reduced proliferation of PMSCs in CEE, we compared the total protein contents of CEE, FBS, and HS (Fig. 1C). CEE had the lowest total protein content, whereas HS had the highest (*P* < 0.01). The protein patterns of HS, FBS, and CEE were assessed by electrophoretic separation (Fig. 1D). Albumin, which accounts for approximately 60% of total serum protein and plays a key role in supporting cell growth [15], was markedly reduced in CEE compared to FBS and HS. Nevertheless, CEE showed several narrow bands, ranging from 15 to 42 kDa. To compensate for the low proliferation rates of CEE, the effects of supplementing 20% and 30% CEE with 1%, 3%, and 5% FBS or HS were evaluated for their impact on cell viability and proliferation (Fig. S3). Given that CEE concentrations above 30% negatively affected PMSC proliferation, we selected 20% and 30% CEE as the baseline conditions. Cell viability was assessed using the CCK-8 assay (Fig. S3A). Among all CEE-supplemented groups, the 20% CEE + 5% HS group showed the highest cell viability at 72 h, which was still lower than that of the 15% FBS group. However, morphological analysis showed that the 20% CEE + 5% HS group exhibited a cell growth rate comparable to that of the 15% FBS group (Fig. S3B). In addition, the FBS supplementation impaired the attachment and morphological maturation of PMSCs (Fig. S3C). To compare cell proliferation rates more precisely among the 15% FBS, 20% CEE, and 20% CEE + 5% HS groups, the total number of cells was counted at 24, 48, and 72 h (Fig. 1E). The mean number of cells was significantly higher in the 20% CEE + 5% HS group (*P* < 0.01) than in the 15% FBS group. Moreover, cells in the 20% CEE + 5% HS group maintained an elongated spindle-like shape and reached over 90% confluency faster than those in the 15% FBS group, with myotube formation observed after 72 h (Fig. 1F). Wound healing assays further confirmed that PMSCs cultured in 20% CEE + 5% HS showed greater migration toward the wound area than those in the 15% FBS group (Fig. 1G).

Next, we compared the apoptotic responses of PMSCs under 15% FBS, 20% CEE, or 20% CEE + 5% HS (Fig. 1H). The 20% CEE group exhibited a significantly lower (*P* < 0.01) live cell and increased early and late apoptosis and necrosis ratios than the 15% FBS and 20% CEE + 5% HS groups (Fig. 1J). The addition of 5% HS to 20% CEE significantly alleviated these effects, resulting in increased cell survival and reduced apoptosis and necrosis compared with 20% CEE alone. In addition, cell cycle analysis revealed significant differences in the distribution of PMSCs among the G0/G1, S, and G2/M phases, depending on the culture conditions (Fig. 1I). The 20% CEE group exhibited a significant increase in the G0/G1 phase and a marked reduction in the S and G2/M phases (Fig. 1K). In contrast, the 20% CEE + 5% HS group showed a reduced G0/G1 population and restored the S phase proportion to a level comparable to that of the 15% FBS group (*P* < 0.01). To further assess proliferative capacity, we performed KI67 immunofluorescent staining (Fig. 1L). The KI67 staining quantification results showed that the 20% CEE + 5% HS (*P* < 0.01) had a higher ratio of proliferating cells compared to both 15% FBS and 20% CEE (Fig. 1M).

### CEE modulates the expression of myogenic regulators in PMSCs

**Fig. 2.**
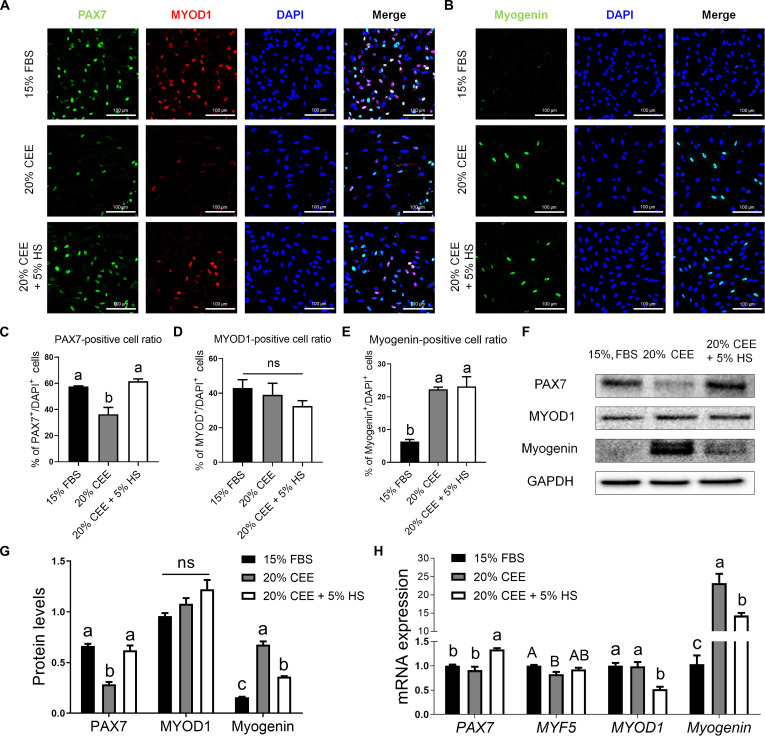
Expression of myogenic transcription factors in PMSCs according to PM composition. (A) Immunofluorescent staining results for PAX7 (green), MYOD (red), and DAPI (blue). (B) Immunofluorescent staining results for Myogenin (green) and DAPI (blue). (C to E) Percentages of PAX7, MYOD1, and Myogenin-positive cells. All percentages were determined by counting PAX7, MYOD, and Myogenin-positive cells in relation to the total number of DAPI-positive cells. (F) Protein expression levels of PAX7, MYOD1, Myogenin, and GAPDH in PMSCs under different culture conditions. (G) PAX7, MYOD, and Myogenin protein expression levels were normalized to GAPDH levels. (H) mRNA expression levels of *PAX7*, *MYF5*, *MYOD1*, and *Myogenin* in PMSCs. *n* = 3. All values are represented as means ± SE. ^a-c^Different superscripts represent statistically significant differences (*P* < 0.01). ^A-B^Different superscripts represent statistically significant differences (*P* < 0.05). ns, nonsignificant.

The expression patterns of PAX7, MYOD1, and Myogenin in PMSCs cultured under 3 different conditions were assessed using immunofluorescence staining (Fig. 2A and B). PAX7 expression in the 20% CEE + 5% HS group was comparable to that in the 15% FBS group, whereas the 20% CEE group showed a significant decrease (*P* < 0.01) (Fig. 2C). No significant differences were observed in the MYOD1-positive cell ratios among the groups (Fig. 2D). The percentage of Myogenin-positive cells was significantly higher (*P* < 0.01) in both the 20% CEE and 20% CEE + 5% HS groups than in the 15% FBS group (Fig. 2E). We also performed western blotting to compare the protein levels in PMSCs according to the medium composition (Fig. 2F). The 15% FBS and 20% CEE + 5% HS groups exhibited similar PAX7 expression levels (Fig. 2G). The expression of PAX7 was lowest in the 20% CEE group (*P* < 0.01). MYOD1 expression levels were not significantly different among the groups. Myogenin protein levels were higher in both the 20% CEE and 20% CEE + 5% HS groups than in the 15% FBS group, with the highest levels observed in the 20% CEE group (*P* < 0.01). Subsequently, we analyzed the mRNA expression levels of *PAX7*, *MYF5*, *MYOD1*, and *Myogenin* using RT-qPCR (Fig. 2H). *PAX7* expression was significantly higher (*P* < 0.01) in the 20% CEE + 5% HS group. The highest expression (*P* < 0.05) of *MYF5* was observed in 15% FBS, and the lowest in 20% CEE. *MYOD1* expression was lowest in the 20% CEE + 5% HS group. The 20% CEE group showed the highest Myogenin expression level (*P* < 0.01), followed by the 20% CEE + 5% HS group, while the 15% FBS group showed the lowest Myogenin expression level. To determine whether these effects originate from CEE or HS, we analyzed a group cultured with 5% HS alone (Fig. S4). Cells in the 5% HS groups exhibited an elongated morphology and reduced proliferation capacity (Fig. S4A). Moreover, the mRNA expression level of *Myogenin* was significantly lower than that of the CEE-treated group (Fig. S4B). Notably, up-regulation of *PAX7* expression was observed only in the 20% CEE + 5% HS condition.

### CEE affects oxidative metabolism and self-renewal via FOXO/NOTCH axis

**Fig. 3.**
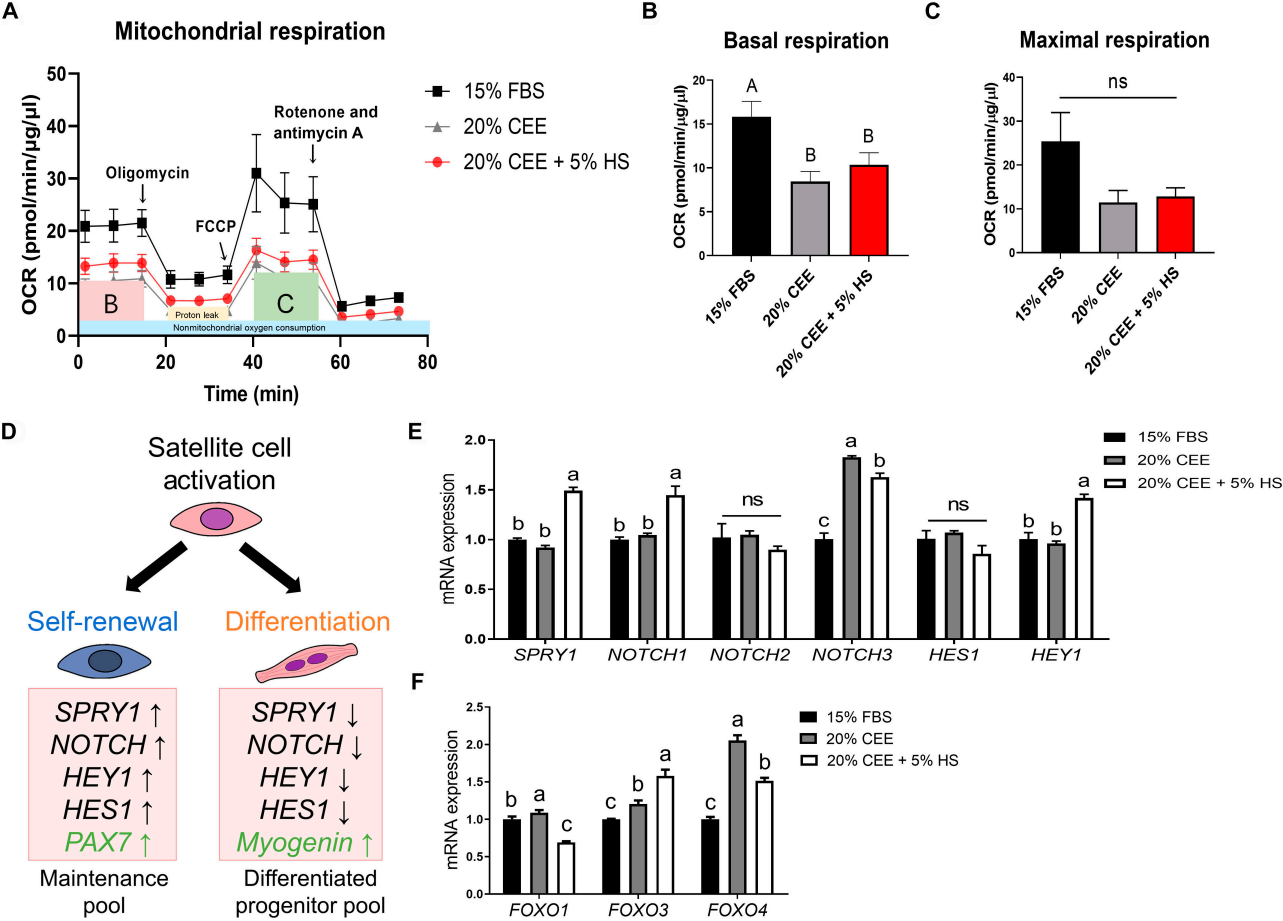
Comparison of OCRs in PMSCs based on the composition of PM culture. (A) The OCR was measured using the Seahorse XFp Analyzer. (B) Basal respiration and (C) maximal respiration were calculated based on the OCR. (D) Diagram showing gene expression patterns associated with the myogenic program in SCs. (E and F) mRNA expression of cell-intrinsic factors that play a role in SC quiescence and self-renewal. *n* = 3. All values are represented as means ± SE. ^A-B^Different superscripts represent statistically significant differences (*P* < 0.05). ^a-c^Different superscripts represent statistically significant differences (*P* < 0.01).

Metabolism determines the state of stem cells and plays a role in regulating their fate [16,17]. To better understand how CEE influence energy metabolism in PMSCs, the OCR, which reflects the activity of cellular oxidative phosphorylation (OXPHOS), was analyzed (Fig. 3A). Under normal conditions, basal respiration is an indicator of mitochondrial OCR, which is necessary for the normal production of cellular energy. The basal respiration levels in the 20% CEE and 20% CEE + 5% HS groups were significantly lower (*P* < 0.05) than those in the 15% FBS group (Fig. 3B). In contrast, maximum respiration is defined as the peak rate of oxygen consumption achieved by the cells after carbonyl cyanide *p*-trifluoromethoxyphenylhydrazone treatment, a compound that disrupts the mitochondrial membrane potential and drives maximal electron transport. No significant differences were observed in the maximal respiration among the groups (Fig. 3C).

To gain deeper insight into the cellular state of PMSCs, the mRNA expression levels of genes involved in quiescence and self-renewal were investigated the mRNA expression levels of genes involved in quiescence and self-renewal (Fig. 3D). The genes associated with stem cell homeostasis, including quiescence maintenance and self-renewal regulation (*SPRY1*, *NOTCH1*, *NOTCH3*, and *HEY1*), were significantly higher (*P* < 0.01) in the 20% CEE + 5% HS group than in the other groups, whereas 15% FBS and 20% CEE showed similar expression levels of *SPRY1*, *NOTCH1*, and *HEY1*. The *NOTCH3* expression was significantly higher in the 20% CEE group (*P* < 0.01) (Fig. 3E). Next, we investigated whether the addition of CEE influenced forkhead box O (*FOXO*) gene expression, which is involved in stem cell homeostasis and regulates the NOTCH signaling pathway (Fig. 3F) [18,19]. In the 20% CEE group, the expression levels of *FOXO* genes were elevated compared to those in the 15% FBS group, with *FOXO1* and *FOXO4* showing the highest expression among the 3 groups (*P* < 0.01). In contrast, the 20% CEE + 5% HS group showed significantly higher *FOXO3* and *FOXO4* expression levels relative to the 15% FBS group, whereas *FOXO1* expression was lower (*P* < 0.01). On the other hand, unlike the 20% CEE + 5% HS group, HS-only condition did not exhibit any up-regulation of FOXO/NOTCH target genes, indicating that these signaling pathways are primarily activated by CEE (Fig. S4C and D).

### Comparison of transcriptomic profiles of PMSCs cultured in FBS- and CEE-based PM

**Fig. 4.**
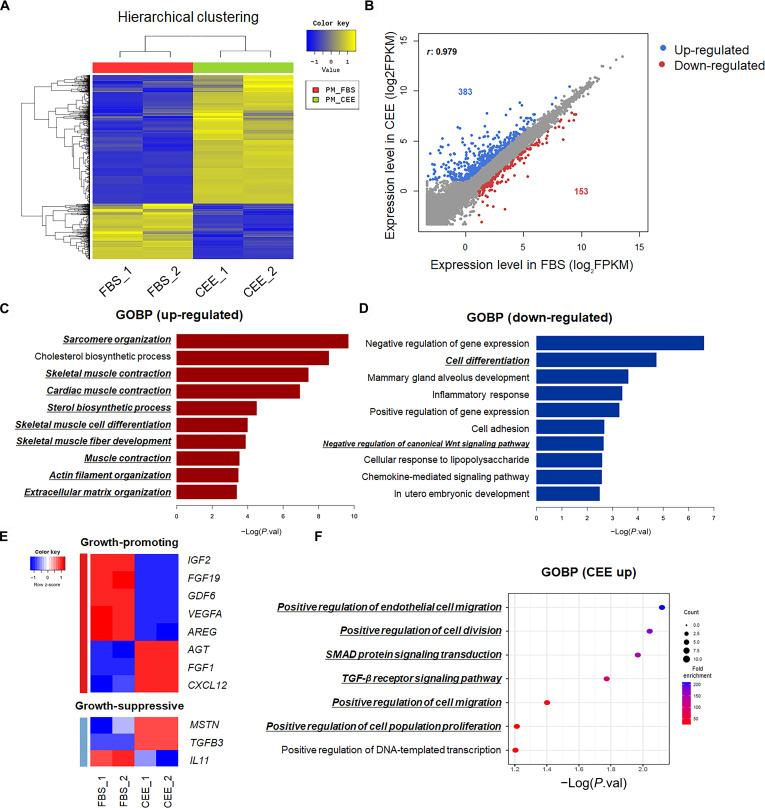
Transcriptomic analysis of PMSCs cultured in 15% FBS or 20% CEE + 5% HS PM. (A) Heatmap of hierarchical clustering based on DEGs in PMSCs according to culture conditions. (B) The scatter plot shows significantly up-regulated and down-regulated genes in the 20% CEE + 5% HS group. (C) GOBPs enriched in up-regulated genes (D) down-regulated genes in the 20% CEE + 5% HS group. (E) Heatmap of DEGs associated with the GO term growth factor activity (GO:0008083). (F) GOBP and KEGG pathway enrichment analysis of up-regulated growth factor activity genes under 20% CEE + 5% HS (CEE). *n* = 2. DEGs were identified on the basis of FC ≥ 2, *P* < 0.05, and FPKM ≥ 2 and were clustered into 2 groups based on expression profiles. All enrichment analyses were performed using DAVID, and the results were visualized using R software.

To investigate the transcriptomic alterations induced by culture in 20% CEE + 5% HS, we performed RNA-seq on PMSCs cultured in either 15% FBS or 20% CEE + 5% HS. Hierarchical clustering analysis revealed distinct transcriptomic expression patterns between the 2 groups, indicating that CEE induced significant changes in gene expression (Fig. 4A). A total of 536 DEGs were identified, of which 383 were up-regulated and 153 were down-regulated under the 20% CEE + 5% HS condition (FC ≥ 2, *P* < 0.05, FPKM ≥ 2) (Fig. 4B). To characterize the biological significance of the DEGs, we conducted GO biological process (GOBP) analysis. The up-regulated genes in the 20% CEE + 5% HS group were significantly enriched in sarcomere organization, skeletal muscle contraction, skeletal muscle cell differentiation, and skeletal muscle fiber development (Fig. 4C). These terms are primarily related to myogenic differentiation processes. However, several differentiation-related terms were also identified as down-regulated (Fig. 4D), such as cell differentiation and negative regulation of the canonical Wnt signaling pathway.

Given that CEE contains various growth factors [7], we sought to determine whether the differential expression observed in PMSCs cultured in 20% CEE + 5% HS media was associated with the regulation of growth-factor-related genes. To investigate this, we analyzed a set of DEGs annotated with the GO term “growth factor activity” (GO:0008083). The 20% CEE + 5% HS group showed a distinct expression pattern compared to the FBS group, indicating a distinct transcriptional response (Fig. 4E). Notably, growth promotion (*AGT*, *FGF1*, and *CXCL12)* and growth suppression (*MSTN* and *TGFB3*) were up-regulated in the 20% CEE + 5% HS group. To further assess the biological implications of the up-regulated genes in the 20% CEE + 5% HS group, we performed GOBP analysis (Fig. 4F). The 20% CEE + 5% HS group specifically enriched cell migration, proliferation, and transforming growth factor-β (TGF-β) receptor signaling-pathway-related genes. In addition, the transcriptional response to growth factor stimulation was examined by analyzing genes classified under the “response to growth factor” GO term (GO:0070848), and heatmap analysis revealed distinct expression patterns in the FBS and 20% CEE + 5% HS groups (Fig. S5A). GOBP analysis revealed that the genes up-regulated in the 20% CEE + 5% HS group were associated with the cellular response to TGF-β stimulus, skeletal muscle cell differentiation, and negative regulation of cell population proliferation (Fig. S5B). In addition, KEGG enrichment analysis of up-regulated genes in the 20% CEE + 5% HS group revealed significant enrichment of the mitogen-activated protein kinase, TGF-β, and phosphatidylinositol 3-kinase–Akt signaling pathways (Fig. S5C). Therefore, distinct growth factor stimulation occurs in the 20% CEE + 5% HS medium compared to that observed in the 15% FBS medium, leading to cellular characteristics that differ from those observed under FBS conditions.

### Myogenic differentiation of PMSCs using CEE

**Fig. 5.**
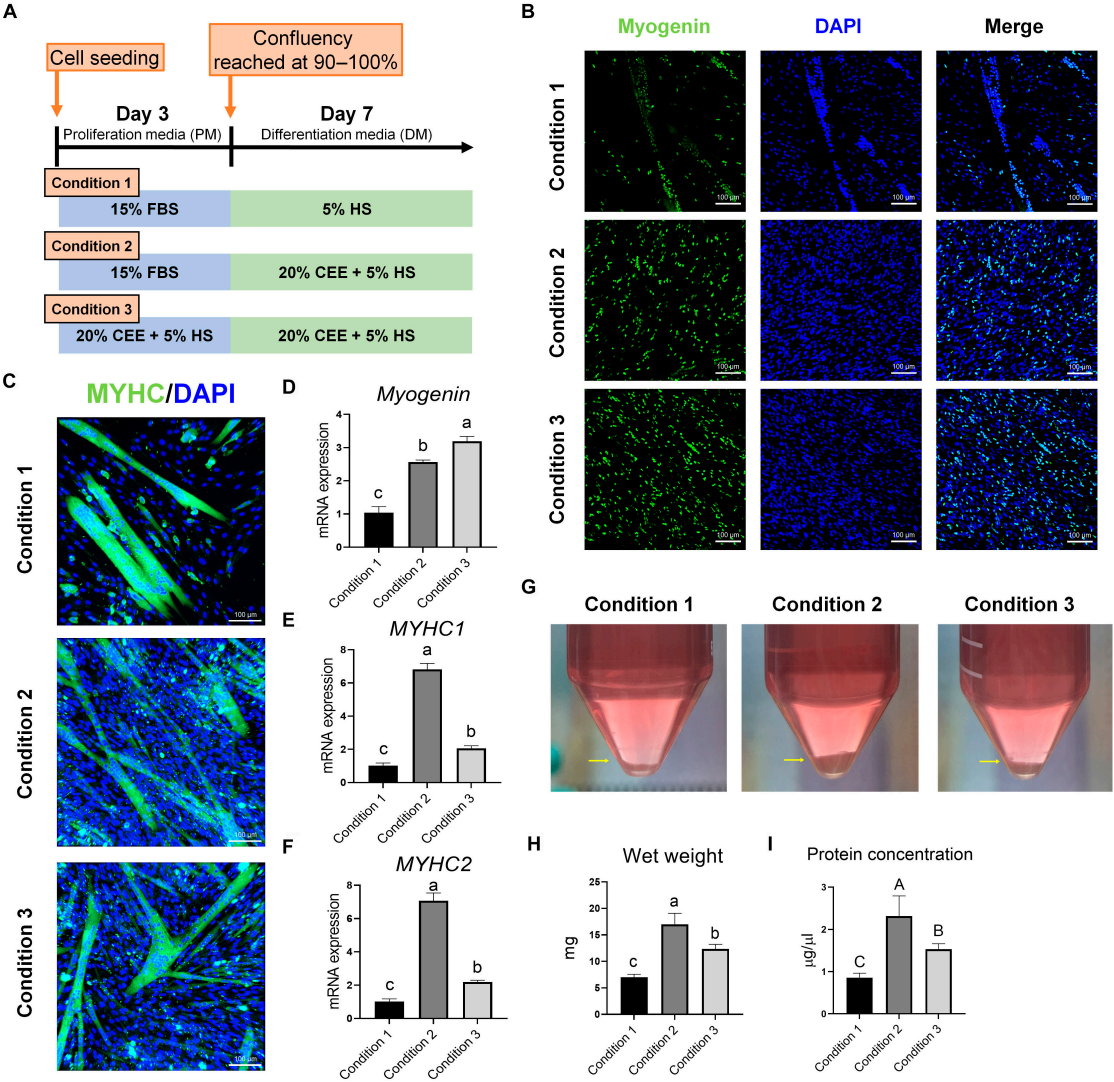
Differentiation of PMSCs in 20% CEE + 5% HS medium. (A) Schematic representation of differentiation experiments under different media conditions. (B) Immunofluorescent staining images of Myogenin (green) and DAPI (blue) after 3 d in DM. (C) Three-dimensional Z-stack visualization of MYHC/DAPI-stained myotubes. (D to F) mRNA expression levels of *Myogenin*, *MYHC1*, and *MYHC2*. (G) Representative images of the final sample amounts after 7 d of culture under different DM conditions. (H) Wet weight and (I) total protein concentration of each group. *n* = 3. All values are represented as the means ± SE. ^a-c^Different superscripts represent statistically significant differences (*P* < 0.01). ^A-C^Different superscripts represent statistically significant differences (*P* < 0.05).

Transcription factor analysis revealed that PMSCs cultured in 20% CEE + 5% HS expressed both PAX7 and Myogenin, indicating that the SCs were undergoing both proliferation and early differentiation (Fig. 2). Therefore, effective differentiation conditions were investigated using the 20% CEE + 5% HS culture phase (Fig. 5A). Condition 1 refers to differentiation with 5% HS, which was used as the control in accordance with our previous study [14]. Under condition 2, only the differentiation phase was replaced with 20% CEE + 5% HS, whereas under condition 3, 20% CEE + 5% HS was used throughout both proliferation and differentiation. After reaching a confluency of 90% to 100%, PMSC differentiation was induced in each DM, and myotube formation was observed for 7 d. To evaluate myogenic differentiation efficiency under CEE treatment, we conducted immunofluorescence staining for Myogenin (Fig. 5B). Conditions 2 and 3, which used 20% CEE + 5% HS during differentiation, exhibited robust Myogenin expression. Subsequently, differentiation was assessed using immunofluorescence staining for MYHC. However, the multilayer structures observed under conditions 2 and 3 interfered with MYHC detection using confocal images (Fig. S6A). To improve visualization, we acquired Z-stack images, which enabled a clearer detection of layered MYHC (Fig. 5C and Movies S1 and S2). MYHC-positive myotubes were detected under all conditions, whereas the total cell numbers were notably higher under conditions 2 and 3 than under condition 1. Furthermore, by day 7 of differentiation, condition 1 failed to maintain myotubes, whereas conditions 2 and 3 continuously formed a multilayer structure while maintaining the underlying myotubes (Fig. S6B). Subsequently, the gene expression of differentiation markers in the differentiated PMSCs from each group was examined (Fig. 5D to F). *Myogenin* expression was significantly higher (*P* < 0.01) under condition 3 and lowest under condition 1 (Fig. 5D). The highest expression levels of *MYHC1* and *MYHC2* were observed under condition 2, whereas condition 1 exhibited the lowest expression levels (*P* < 0.01) (Fig. 5E and F). Furthermore, condition 2 yielded the highest amount of harvested samples, whereas condition 1 yielded the lowest (Fig. 5G). Consistently, wet weight and protein concentration were significantly higher under conditions 2 and 3 compared with condition 1, with condition 2 showing the highest levels (Fig. 5H and I). Therefore, condition 2, which incorporated a 20% CEE + 5% HS culture phase following proliferation in FBS-based medium, most effectively supported the myogenic differentiation of PMSCs.

### DM containing 20% CEE + 5% HS promotes cell sheet formation

**Fig. 6.**
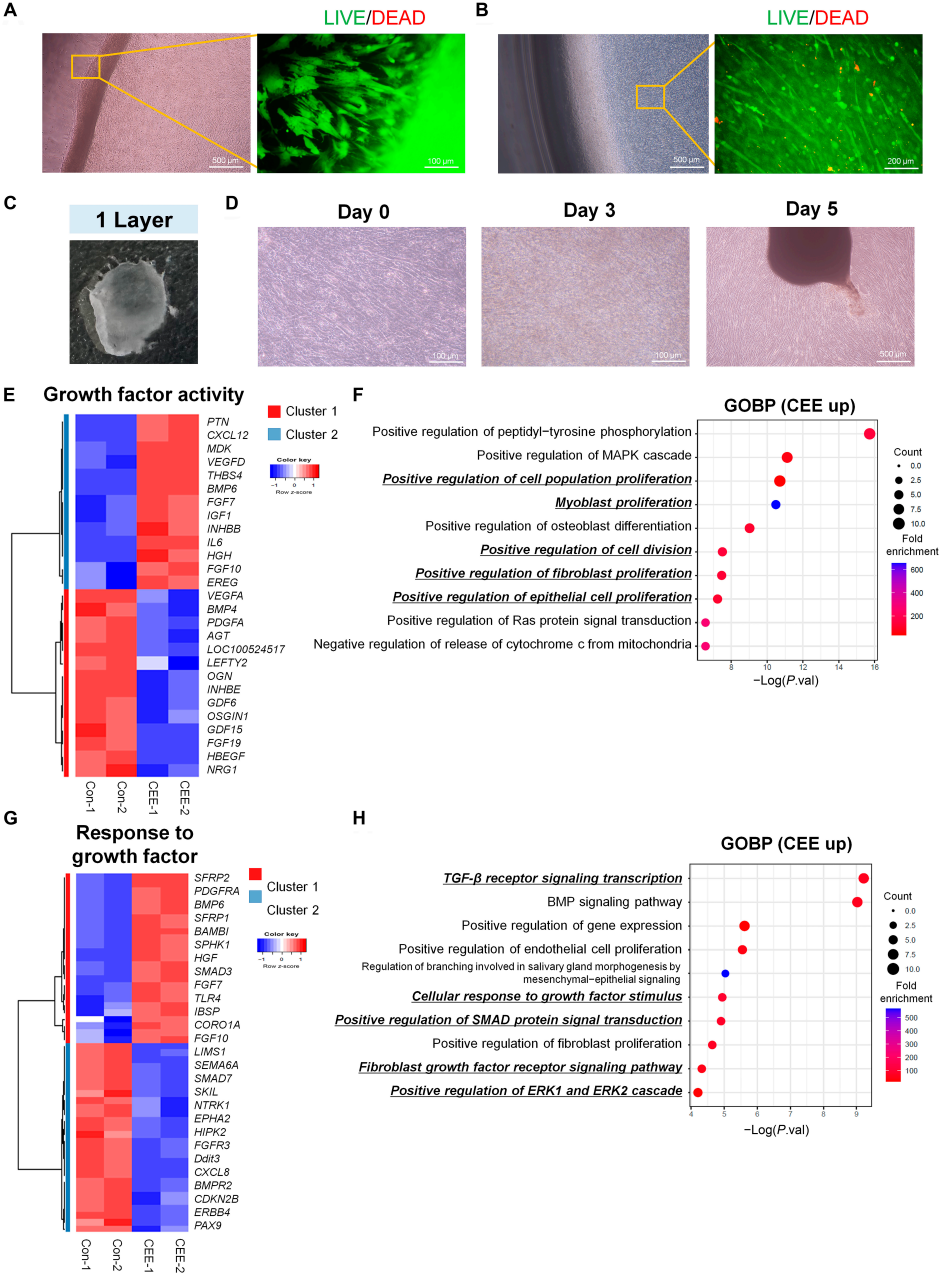
Cell sheet formation and transcriptomic profiling of PMSCs under 20% CEE + 5% HS differentiation conditions. (A and B) Bright-field and LIVE/DEAD staining images of PMSCs cultured in 20% CEE + 5% HS for 5 d. Viable and dead cells appeared green and red, respectively. *n* =3. (C) Appearance of the detached cell sheet after transfer to a new culture dish. (D) Morphological images of the reattached cell sheet on days 0, 3, and 5. All dishes were used for reattachment and precoated with 0.15% gelatin. (E) Heatmap of DEGs associated with the GO term growth factor activity (GO:0008083). (F) GOBP enrichment analysis of up-regulated growth factor activity genes under 20% CEE + 5% HS (CEE). (G) Heatmap of DEGs associated with the GO term response to growth factor (GO:0070848). (H) GOBP enrichment analysis of up-regulated response to growth factor genes in the 20% CEE + 5% HS group (CEE). *n* = 2. DEGs were identified on the basis of FC ≥ 2, *P* < 0.05, and FPKM ≥ 2.

Transcriptomic analysis indicated that TGF-β signaling was regulated in PMSCs cultured in PM containing 20% CEE + 5% HS, with *TGFB3* significantly up-regulated (Fig. 4E and F and Fig. S5). This signaling pathway is associated with the promotion of ECM synthesis [20]. Therefore, we investigated whether the 20% CEE + 5% HS modulated the expression of ECM-related genes in PMSCs. KEGG pathway mapping revealed that the 20% CEE + 5% HS PM culture conditions effectively up-regulated genes associated with collagen and laminin but had no significant effect on fibronectin expression (Fig. S7). Based on the observed up-regulation of ECM-related genes under the 20% CEE + 5% HS condition, we investigated whether this condition supports the formation of muscle cell sheets from PMSCs during differentiation culture. Notably, muscle cell sheets were observed on day 5 of differentiation (Fig. 6A), whereas no such structures appeared under FBS-based conditions. Cell sheets appeared to form through gradual curling and lifting at the dish periphery during differentiation, resulting in a rolled-edge morphology. Importantly, the high confluence associated with cell sheet formation did not negatively affect the cell viability (Fig. 6A and B). The cell sheet construct demonstrated sufficient mechanical durability for manual handling and could be detached as a single cell sheet simply by scraping along the edge without any additional treatment (Fig. 6C). The constructs were transferred to new culture dishes to evaluate the stability of the cell sheets. We assessed the retention and adhesion of the cell sheets by culturing them on dishes with or without a gelatin coating (Fig. S8A). Cell sheets transferred onto noncoated dishes formed aggregates as early as day 1 of culture (Fig. S8B). In contrast, on the 0.15% gelatin-coated dish, the cell sheets adhered to the surface, allowing prolonged maintenance of the sheet construct, with aggregation observed only after 5 d (Fig. 6D). Furthermore, the detached cell sheets retained aligned myotube morphology, indicating that their structural organization was preserved after detachment.

To elucidate the molecular mechanisms underlying cell sheet formation under 20% CEE + 5% HS conditions, we conducted RNA-seq on day 5 after sheet formation, comparing CEE-induced cell sheets with cells differentiated under 5% HS (Con). A heatmap of DEGs demonstrated distinct clustering patterns according to the differentiation conditions (FC ≥ 2, *P* < 0.05, FPKM ≥ 2) (Fig. S9A). Scatter plot analysis revealed that 526 genes were up-regulated and 463 genes were down-regulated in the CEE group compared to the control group. GOBP analysis of CEE-up-regulated genes indicated the enrichment of pathways related to cell proliferation, survival, and angiogenesis (Fig. S9B). Conversely, the down-regulated genes were primarily associated with cell adhesion processes. Furthermore, to determine whether the activation of growth-factor-related pathways observed during proliferation was involved in CEE-induced cell sheet formation, we analyzed the transcriptional response during differentiation was analyzed (Fig. 6E to H). GOBP enrichment analysis of up-regulated genes related to growth factor activity revealed significant enrichment of terms associated with cell proliferation (Fig. 6F). In addition, the GOBP enrichment analysis of up-regulated DEGs associated with the GO term “response to growth factor” revealed a significant enrichment of TGF-β-related signaling pathways, including TGF-β receptor signaling transduction, positive regulation of SMAD protein signal transduction, fibroblast growth factor (FGF) receptor signaling pathway, and positive regulation of *ERK1* and *ERK2* cascades (Fig. 6H). Therefore, growth-factor-responsive pathways, particularly those related to TGF-β signaling, may play a key role in cell sheet formation. In addition, the pathway map revealed a marked up-regulation of collagen-associated genes, whereas key laminin and fibronectin genes were predominantly down-regulated (Fig. S10). This shift in ECM gene expression suggests a transition toward a collagen-enriched matrix environment, which may contribute to enhanced cell–cell and cell–matrix interactions during cell sheet formation.

### Formation of CM patties using multilayered cell sheet constructs via stacking-based fabrication

**Fig. 7.**
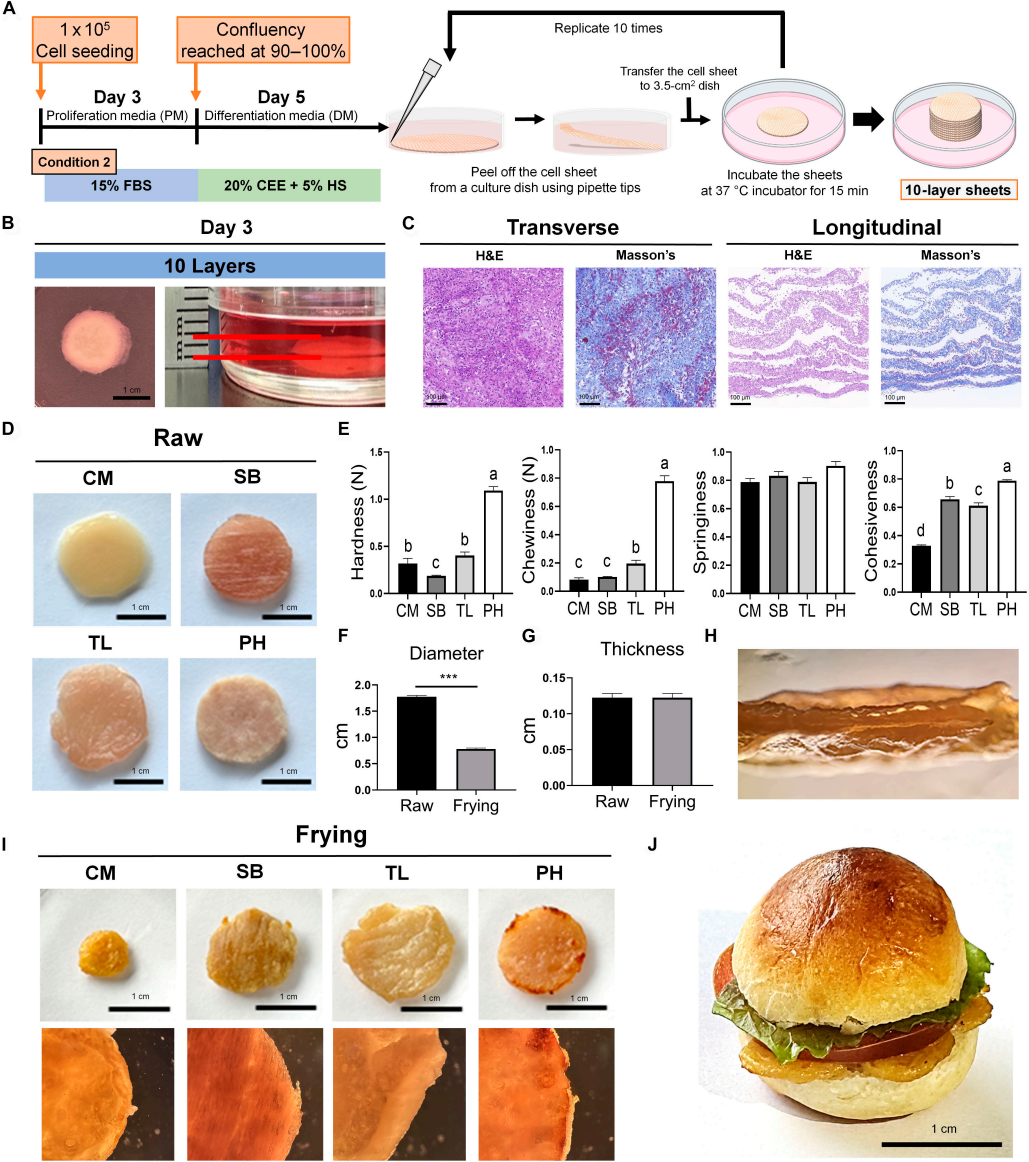
Fabrication and characterization of multilayered cell-sheet-based CM. (A) Schematic illustration of the process for fabricating multilayered cell sheets. After differentiation induction for 5 d in 6-well plates, the cell sheets were peeled off and sequentially layered (10 layers) into 3.5-cm^2^ dishes. (B) Morphological analysis of 10 layered cell sheet constructs was performed on day 3 of extended culture in CEE DM after transfer to 3.5-cm^2^ dishes. (C) Histological analysis of multilayered cell sheet constructs using H&E and Masson’s trichrome staining. In H&E staining, nuclei appear blue to dark purple, whereas the cytoplasm and other tissue components are stained pink. Masson’s trichrome staining showed collagen in blue, nuclei in black, and muscle fibers and cytoplasm in red or pink. (D) Representative images of multilayered cell sheet CM, defrosted SB, TL, and PH. (E) Comparison of hardness, chewiness, springiness, and cohesiveness of raw CM and commercial pork. *n* = 4. ^a-d^Different superscripts represent statistically significant differences (*P* < 0.01). (F and G) Diameter and thickness of CM constructs were measured in the raw state and after frying. *n* = 3. Statistical significance between the 2 groups was determined using Student *t* test. ***P < 0.001. (H) Cross-sectional image of the multilayered CM. (I) Top images show the overall surface morphology of each sample after frying. The bottom images show the microstructures of the same samples observed using a stereoscopic microscope. (J) Representative images of hamburgers constructed using multilayered CM. All values are represented as the means ± SE.

Various scaffold-free strategies have been developed to mimic tissue-like structures. Among these, cell sheet engineering has gained attention due to its ability to generate dense ECM-rich constructs without exogenous scaffolds [13]. These well-organized cell-sheet-based tissues secrete their own ECM, contributing to the development of CM with structural and textural properties that closely resemble those of conventional meat [12]. In this study, we constructed a 10-layered muscle tissue by sequentially stacking myogenic cell sheets generated under the 20% CEE + 5% HS differentiation conditions (Fig. 7). To optimize the culture period for harvesting laminated multilayered cell sheets, we assessed morphology, diameter, and thickness using small-scale CM constructs (5 × 10^4^ cells per sheet) (Fig. S11A). On gelatin-coated surfaces, stacked cell sheets exhibited stable attachment, as well as proliferation and migration (Fig. S11B). They achieved maximal size by day 3, which has been identified as the optimal duration for harvesting (Fig. S11C). Subsequently, 10 cell sheets (1 × 10^5^ cells per sheet) were stacked and harvested on day 3 to produce larger multilayered cell sheet constructs (Fig. 7A and B). No delamination or structural failure was observed during the culture period. To better understand the composition and cellular organization of the 10-layered CM constructs, we performed histological staining using H&E and Masson’s trichrome staining (Fig. 7C). The CM maintained a well-organized layered architecture and showed abundant ECM components, similar to the small-scale prototype CM (Fig. S11D and E). To evaluate the texture, we performed TPA on raw 10-layered CM, defrosted SB, TL, and PH (Fig. 7D). PH exhibited the highest values for hardness, chewiness, and cohesiveness, which were significantly different from those of the other samples (Fig. 7E). The hardness of CM was comparable to that of TL and significantly greater than that of SB (*P* < 0.01). In terms of chewiness, CM was similar to that of SB but lower than that of TL (*P* < 0.01). Among all the samples, CM exhibited the lowest cohesiveness. No significant differences were observed in springiness across the groups, indicating comparable elastic properties of the materials. The CM was pan-fried to evaluate its structural and physical integrity during cooking. After frying, the diameter of the CM significantly decreased, whereas its thickness remained unchanged (Fig. 7F and G). Furthermore, the layered cell sheet structure was preserved after heating, demonstrating the structural stability of CM (Fig. 7H). In addition, fried CM exhibited Maillard browning and surface characteristics (including texture, color, and appearance) closely resembling those of fried commercial pork products (Fig. 7I). Finally, a prototype hamburger was constructed using CM to demonstrate its feasibility for application in real food products (Fig. 7J).

## Discussion

To lower CM production costs, it is imperative to develop affordable culture media that can effectively support muscle cell proliferation and differentiation. Although FBS is commonly used in the majority of cell culture systems due to its abundance of proteins and small-molecule metabolites [4,5], it remains expensive. The total cost of the 20% CEE + 5% HS medium was about 45,000 KRW (USD 31) per liter. In comparison, the conventional 15% FBS-based medium costs around 300,000 KRW (USD 205) per liter, meaning that the CEE-based formulation is roughly 85% cheaper. Therefore, the 20% CEE + 5% HS is an economical and promising functional alternative for supporting the proliferation and differentiation of PMSCs. This medium not only promoted self-renewal, proliferation, and differentiation but also enhanced ECM production in PMSCs. These properties enable the simple and efficient generation of myotube cell sheets. CM prototypes were successfully constructed by stacking these sheets. To the best of our knowledge, this is the first study to demonstrate the potential of CEE not only as an FBS functional substitute for PMSCs culture but also as a practical and scalable component in the engineering of CM constructs.

In this study, replacing FBS with CEE alone in PMSC cultures reduced cell viability and induced morphological changes. These effects are likely associated with substantial compositional differences in proteins and bioactive molecules among FBS, HS, and CEE, as revealed by protein pattern analysis. Notably, both HS and FBS exhibited a prominent protein band at approximately 60 kDa, corresponding to the molecular weight of albumin, whereas CEE displayed a relatively weak band. Albumin induces mammalian cell proliferation and interacts with intracellular signaling molecules, such as insulin and epidermal growth factor, contributing to enhanced mammalian cell proliferation and survival [15]. Therefore, the reduced proliferation in CEE cultures may result from its distinct protein composition, including lower albumin. This deficiency in CEE was compensated by adding HS, a cheaper serum than FBS, costing about 21.7 KRW (USD 0.01) per milliliter versus 1,644 KRW (USD 1.12) per milliliter for FBS. The combination of 20% CEE and 5% HS was the optimal condition for effectively supporting PMSC cultures.

Although the CEE contained relatively low levels of albumin, multiple distinct protein bands were observed in the 15- to 45-kDa range. Christman et al. [6] suggested that all essential factors required for egg development after fertilization are inherently present within the embryo, implying that CEE may contain numerous growth factors. Based on their molecular weights, these protein bands may correspond to growth factors. RNA-seq further demonstrated the differential expression of growth-factor-related genes between the 20% CEE + 5% HS and control groups. In particular, in 20% CEE + 5% HS group, PMSCs showed up-regulation of CXCL12, FGF, and TGF-β3. CXCL12, a member of the CXC chemokine family, stimulates cell proliferation, facilitates myoblast migration, and inhibits myogenic differentiation [21]. In practice, the injection of CXCL12 into muscles can increase muscle mass and decrease fibrosis. FGFs are a large family of polypeptide growth factors that are present in chick embryos [22]. They play a vital role in the self-renewal and maintenance of skeletal muscle SCs [23]. In particular, FGF1, FGF2, and FGF6 promote proliferation and asymmetric division and inhibit differentiation [23,24]. TGF-β is known to up-regulate ECM-related genes and inhibit the myogenic differentiation of myoblasts [20]. Three types of TGF-β isoforms, TGF-β1, TGF-β2, and TGF-β3, exhibit distinct physiological effects despite structural similarity [25,26]. TGF-β1 and TGF-β2 are primarily associated with profibrotic activity and induce fibrotic phenotypes. In contrast, TGF-β3 exerts antifibrotic effects [27]. Collectively, these results indicate that CEE contains a variety of growth-promoting factors despite its low albumin content, as evidenced by the presence of multiple low-molecular-weight proteins and the up-regulation of growth-factor-related genes, such as CXCL12, FGF, and TGF-β3. These factors play key roles in self-renewal, cell proliferation, myoblast activity, and ECM regulation, suggesting that the CEE medium can effectively support PMSC growth and function.

SCs undergo myogenic differentiation through the temporal expression of specific myogenic transcription factors [28]. All quiescent and activated SCs express PAX7. When quiescent SCs are activated, they exhibit a PAX7^+^/MYOD^+^/Myogenin^−^ profile, which is characteristic of myoblasts [29,30]. When differentiation signals persist, PAX7 is down-regulated, and differentiation is initiated, accompanied by a subsequent increase in Myogenin expression [31]. In contrast, the induced up-regulation of PAX7 in SCs and down-regulation of MYOD promote the self-renewal and maintain quiescence [32]. Therefore, the expression patterns of PAX7, MYOD, and Myogenin serve as indicators distinguishing the quiescence, activation, self-renewal, and differentiation of SCs [32,33]. Furthermore, owing to the mutual exclusivity of PAX7 and Myogenin, they are rarely coexpressed [34]. However, the 20% CEE + 5% HS group showed high PAX7 expression while simultaneously showing elevated expression of Myogenin. RNA-seq showed that while the GOBP enrichment of up-regulated genes reflected active muscle differentiation, some differentiation-related terms were simultaneously down-regulated. This paradoxical profile suggests cellular heterogeneity within the population, likely reflecting asymmetric division that gives rise to both committed and self-renewing progeny. Cell fate is governed by 2 types of cell division, symmetric and asymmetric [30]. Asymmetric division generates 1 committed cell (PAX7^+^/MYF5^+^, capable of becoming differentiated cells) and 1 self-renewal cell (PAX7^+^/MYF5^−^, contributing to the maintenance of homeostasis) [30,35]. The observed myogenic profiles under 20% CEE and 5% HS indicate asymmetric division that supports both self-renewal and differentiation within the cell population.

To gain deeper insight into the current state of PMSCs, we compared OCR rates and self-renewal associated gene expression. During the transition between quiescence, activation, and differentiation, the metabolic activity of muscle SCs undergoes changes [16,36]. Upon activation, muscle SCs undergo transcriptional changes, accompanied by an increase in OXPHOS [36]. In contrast, quiescent SCs exhibit a low metabolic rate. Abreu and Kowaltowski [37] reported that SCs isolated from exercised mice showed elevated expression of self-renewal and quiescent markers, along with reduced OCR rates compared to normal SCs. Consistent with SCs from exercised mice, PMSCs cultured in 20% CEE + 5% HS exhibited reduced basal respiration compared with FBS-based conditions. In addition, the expression of self-renewal- and quiescence-associated markers, including *PAX7*, *SPRY1*, *NOTCH1*, *NOTCH3*, *HEY1*, *FOXO3*, and *FOXO4*, was up-regulated, whereas *FOXO1* and *MYOD1* expression was down-regulated. SC self-renewal is intricately linked to and regulated by the NOTCH signaling pathway. Upon activation of NOTCH signaling, NOTCH intracellular domain interacts with protein Jκ, leading to the induction of PAX7 and suppression of MYOD expression [30]. The transcription factors FOXO1 and FOXO3 can induce the NOTCH signaling pathway, which is crucial for maintaining the quiescence and self-renewal of muscle SCs [18,19] and regulating asymmetric cell fate decisions [38]. During muscle regeneration, FOXO3 is up-regulated in a reduced growth factor environment, promoting the reentry of SCs into a quiescent state [18]. In addition, FOXO3 promotes SC differentiation during the late stages of myogenesis [38]. FOXO1 suppresses myoblast differentiation and functionally interacts with the NOTCH signaling pathway to modulate the progression of muscle cell differentiation [19]. In our study, PMSCs cultured in 20% CEE + 5% HS showed increased expression of *FOXO3* and decreased expression of *FOXO1*. Notably, among the 3 conditions tested, only 20% CEE + 5% HS led to the up-regulation of NOTCH downstream target gene *HEY1* [30]. These results suggest that the 20% CEE + 5% HS condition promotes the FOXO/NOTCH axis for expansion of self-renewing cell populations and favors asymmetric division, which may account for the observed coexpression of PAX7 and Myogenin.

The group treated with 20% CEE + 5% HS demonstrated enhanced proliferation compared to the 15% FBS group, accompanied by reduced OXPHOS activity. This reduction suggests a metabolic adaptation reminiscent of the Warburg effect, in which rapidly proliferating cells preferentially rely on glycolysis over OXPHOS even under normoxia [39,40]. This shift is observed in highly proliferative cells, including cancer cells, and regenerating SCs [41,42]. Although adenosine triphosphate production is more efficient through OXPHOS, highly proliferative cells often adopt glycolysis as their primary energy source because it produces adenosine triphosphate up to 10 times faster than OXPHOS [42,43]. In addition, pyruvate produced during glycolysis is converted mainly to lactate [42], which accumulates in cells and promotes SC differentiation [44]. Thus, the reduced OXPHOS (basal and maximal respiration) observed in the 20% CEE + 5% HS group reflects a metabolic state that supports both rapid proliferation and priming for differentiation.

This metabolic state also explains why the results of the CCK-8 assay did not fully align with those of direct cell count and morphological assessments. CCK-8 is a colorimetric assay based on the reduction of water-soluble tetrazolium 8 by cellular dehydrogenases, which are predominantly active within the mitochondria [45]. OCR becomes significantly reduced during self-renewal or quiescent states, despite the cells remaining viable [37]. As a result, the low mitochondrial activity in the 20% CEE + 5% HS group may have led to a lower CCK-8 signal, potentially underestimating actual cell viability or proliferation. Therefore, the discrepancy between CCK-8 absorbance and other proliferation indicators may result from the metabolite-dependent nature of the assay, which reflects mitochondrial function rather than total cell number.

We observed successful differentiation of PMSCs into myotubes using the 20% CEE + 5% HS condition as a DM. In addition, the continuous increase in cell number under 20% CEE + 5% HS differentiation conditions led to the formation of densely structured cell sheets, resulting in a higher sample yield than that of the control group. Cell density within a confined area significantly influences skeletal muscle differentiation. High cell densities facilitate the formation of compact and durable structures [12]. Generating large amounts of tissue is crucial for cost reduction in CM production. Therefore, this study demonstrates the potential of CEE to enhance myogenic tissue differentiation and increase overall tissue formation, offering a more efficient approach for scalable CM production. However, differentiation was evaluated only under the 20% CEE + 5% HS condition, and a CEE-only differentiation condition was not included in the present study. This decision was based on preliminary optimization considerations, as serum-derived components are commonly used to support efficient myoblast fusion and stable myotube formation in PMSCs. Accordingly, the differentiation outcomes observed here reflect the performance of CEE within a serum-supported differentiation context rather than the independent effect of CEE alone. Therefore, CEE-specific effects on myogenic differentiation should be interpreted with caution, and further studies will be required to systematically evaluate the differentiation capacity of CEE as a standalone or serum-free supplement. In this regard, the current findings should be understood as evidence supporting the feasibility of CEE-based DM as an optimization strategy to improve or complement conventional differentiation conditions, thereby enabling sustained myogenic differentiation and efficient CM production, rather than as definitive proof of an autonomous differentiation-inducing effect of CEE.

Under 20% CEE + 5% HS differentiation conditions, PMSCs formed layered, sheet-like structures without ECM supplementation. This structural organization was accompanied by the up-regulation of ECM-related genes, suggesting that CEE medium activates signaling pathways conducive to ECM formation. Transcriptomic analysis revealed an increase in the expression of TGF-β3, which stimulates collagen production without inducing a fibrotic phenotype [27]. While collagen genes are up-regulated, fibronectin expression is down-regulated, indicating that ECM remodeling observed under these conditions may not be indicative of fibrosis [20]. Furthermore, we observed the simultaneous up-regulation of key myogenic markers, such as PAX7, MYOG, and MYHC, signifying active myogenesis alongside ECM remodeling.

In engineered tissues, the development and accumulation of ECM components are vital to ensure proper cell alignment, mechanical integrity, and overall functional performance [46]. Cell sheets offer the advantage of maintaining the ECM in a structurally intact form during the harvesting process, resulting in higher ECM content [47]. However, these cell sheets often require prolonged culture periods to accumulate sufficient ECM, making them difficult to handle during downstream processing [12,13]. In this study, the 20% CEE + 5% HS differentiation conditions rapidly promoted the formation of cell sheets with significantly increased tissue yield, total protein content, and ECM accumulation. The multilayered cell-sheet-based approach offers a promising platform for generating structured CM without relying on synthetic scaffolds. By enabling the direct stacking of muscle-derived cell sheets, this method allows controlled tissue assembly while preserving native ECM components and cell–cell interactions. This property supports the formation of stable tissues in scaffold-free constructs and CM [12]. Consistent with this, TPA of the resulting CM revealed that the CEE-derived sheets displayed mechanical properties comparable to those of commercial pork cuts, with a hardness similar to that of TL and chewiness approaching that of SB. Collectively, these findings suggest that CEE-based cell sheets can replicate the texture of real meat through a scaffold-free approach that utilizes only muscle cells, without the need for synthetic or edible scaffolds, providing a cost-effective and streamlined method.

In addition to these structural and functional advantages, the use of CEE may also carry practical environmental and ethical benefits compared with FBS. FBS is obtained from bovine fetuses, which involves substantial animal sacrifice and high greenhouse gas emissions [48]. In contrast, CEE is derived from fertilized chicken eggs at an early embryonic stage, a smaller-scale source that requires fewer resources. CEE can be prepared from fertilized chicken eggs that are not suitable for hatching or consumption, such as surplus or male eggs from poultry facilities. The use of these otherwise discarded biological resources further enhances the sustainability of CEE preparation by reducing waste and utilizing low-value by-products. From an environmental perspective, poultry-egg systems generally show much lower life-cycle burdens, representing the overall environmental impact across production and processing stages, and are estimated to be less than one-third of those associated with cattle-based systems, reflecting their higher resource efficiency [49]. Although CEE remains an animal-derived material, these aspects collectively indicate that CEE-based systems may represent a more sustainable and ethically moderate approach for serum supplementation in CM production.

In conclusion, our results indicate that culturing PMSCs in 20% CEE + 5% HS medium provides an effective functional alternative to traditional FBS-based systems, enabling a substantial reduction in FBS dependency while promoting both self-renewal and differentiation. These processes sustain the SC population and provide a continuous supply of nuclei essential for the formation of myotubes. This is especially beneficial for CM production, where maximizing tissue yield while maintaining cell identity is essential. Notably, CEE supplementation led to a significant up-regulation of ECM-related genes and facilitated the formation of structurally stable cell sheets characterized by elevated protein content and enhanced myogenic marker expression. In addition, unlike previously reported cell sheet engineering methods that rely on temperature-sensitive polymers (such as poly(N-isopropylacrylamide)) or enzymatic treatments for cell harvesting [50]. The 20% CEE + 5% HS medium enables the effortless detachment of intact cell sheets through simple mechanical scraping. This approach reduces structural damage and simplifies the overall process. However, achievable tissue thickness remains limited, and manual stacking restricts scalability. Future study should focus on enhancing automated stacking methods and diffusion-enhancing techniques, such as perfusion-based culture, microchannel integration, and the assembly of prevascularized cell sheet layers. Moreover, efforts should be directed toward identifying essential bioactive factors in CEE that support the development of thicker, more physiologically relevant CM tissues.

## Ethical Approval

All animal experiments were approved by the Institutional Animal Care and Use Committee of Konkuk University (KU25116) and conducted in accordance with national guidelines.

## Data Availability

Data will be made available on request.
