# Supplementary material for: Chicken Embryo Extract Remodeling of Extracellular Matrix Sustains Self-Renewal and Differentiation for Scaffold-Free Cell Sheet Formation
Source: Biomater Res. 2026 Mar 3;30:0332. doi: 10.34133/bmr.0332 (PMC12953922; doi:10.34133/bmr.0332)
Supplement: Supplementary 1 — Figs. S1 to S11 Table S1 Movies S1 to S2 [file bmr.0332.f1.zip › ## Revision_ Biomaterials Research supple file_ver 3.docx]

**SUPPLEMENTARY FIGURES**

**
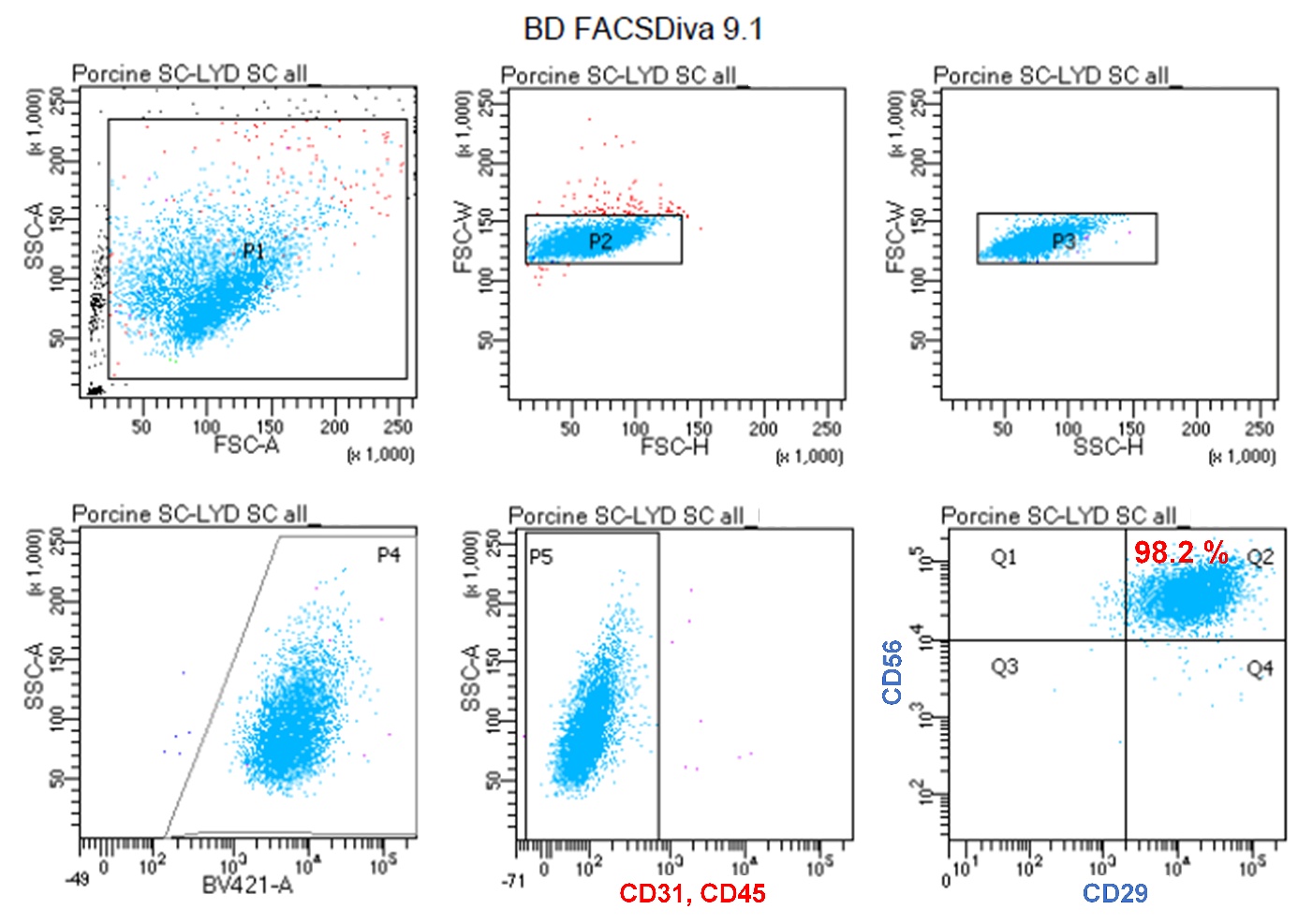
**

**Fig. S1. Purity identification of porcine muscle satellite cells (PMSCs) using specific cell surface markers.** The cells were gated by SSC and FSC prior to gating for CD31, CD45, CD29, and CD56.


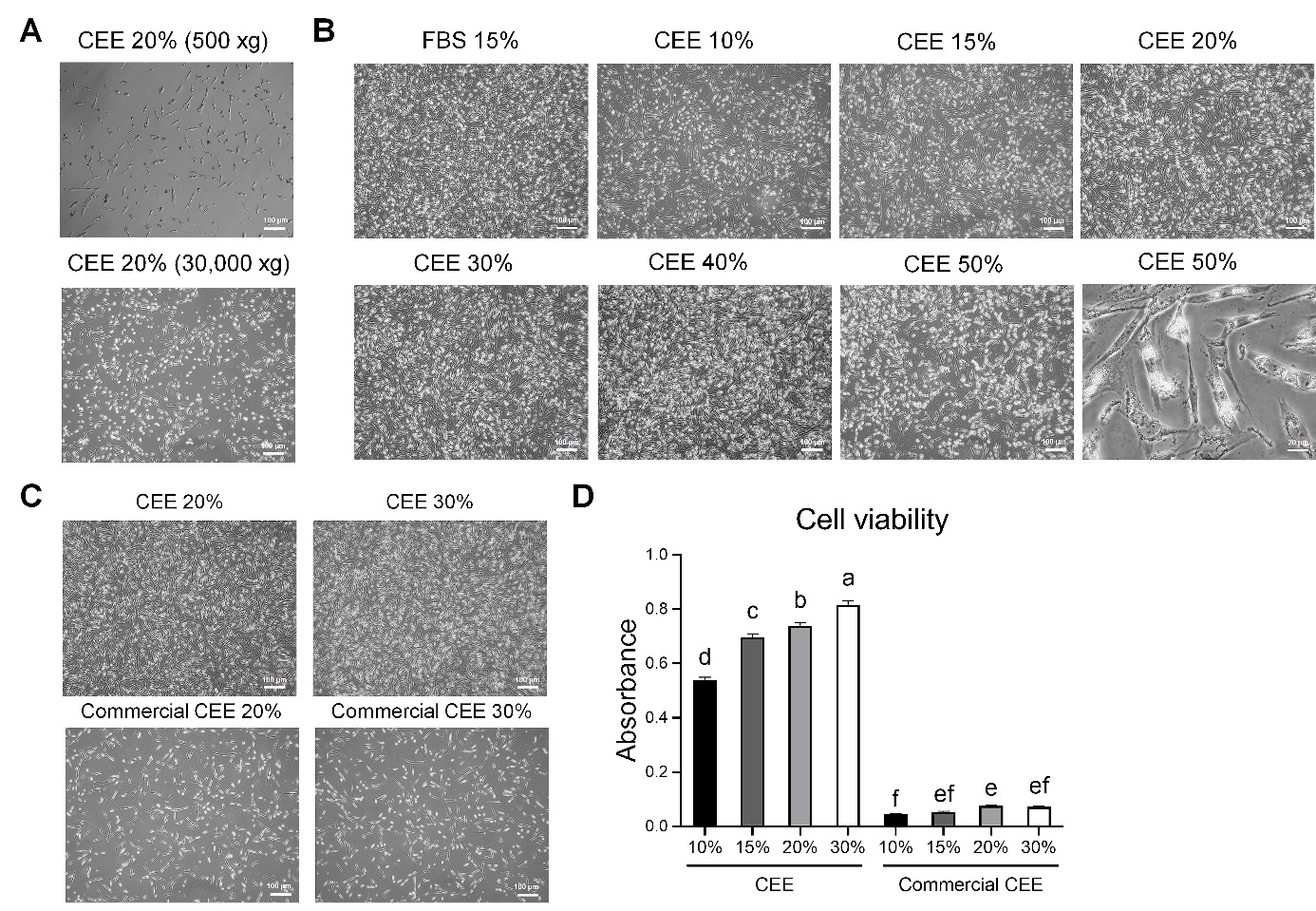


**Fig. S2. Effects of the addition of propidium iodide to the media.** (A) The CEE morphology depends on the centrifugation speed (500 × g and 30,000 × g) used for CEE extraction. (B) Assessment of PMSC morphology after 72 h culture with varied CEE (10, 15, 20, 30, 40, and 50%) concentrations. (C) Comparison of the cell viability and morphology between in-house prepared CEE and commercial CEE. The commercial CEE (Life Science Group Ltd, Sandy, UK) was used for comparison with the self-produced CEE. (D) The cell viability measurements were conducted in six replicates. All values are represented as the mean ± standard error (SE). ^a-d^ Different superscripts represent statistically significant differences (*p* < 0.01). CEE, chicken embryo extract; FBS, fetal bovine serum.


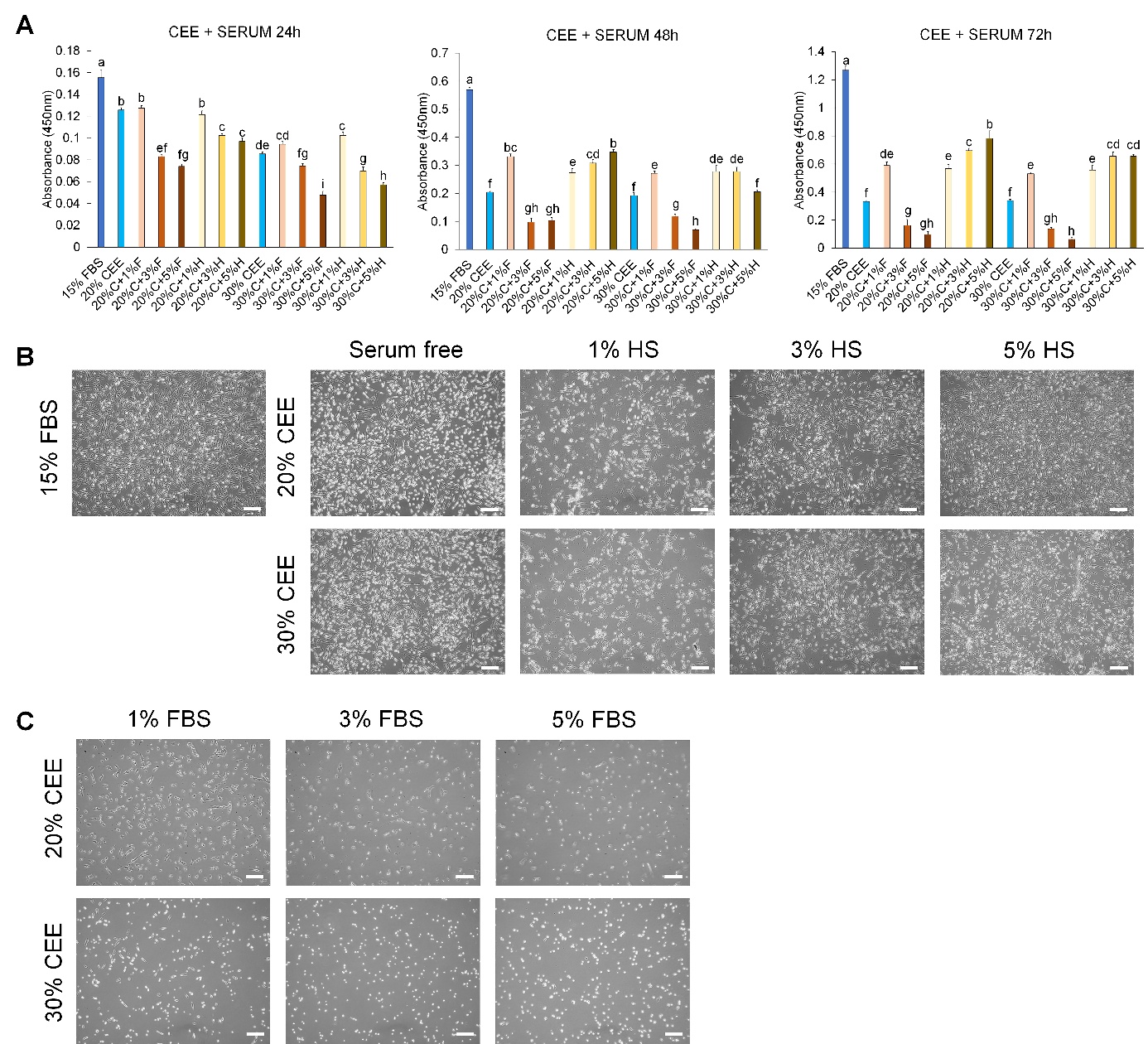


**Fig. S3 Selecting appropriate culture conditions using CEE supplemented with FBS or CEE**. (A) Comparison of cell viability across culture conditions using the cell counting kit-8 (*n* = 6). (B) The morphological analysis was conducted under various culture conditions using CEE supplemented with HS for 48 h. (C) The morphological analysis was conducted under various culture conditions using CEE supplemented with FBS for 24 h. Scale bars: 100 µm (*n* = 3). ^a-i^ Different superscripts represent statistically significant differences (*p* < 0.01). The presence of identical superscripts indicates that there are no significant differences. All values are represented as the mean ± standard error (SE). CEE, chicken embryo extract; FBS, fetal bovine serum; HS, horse serum.


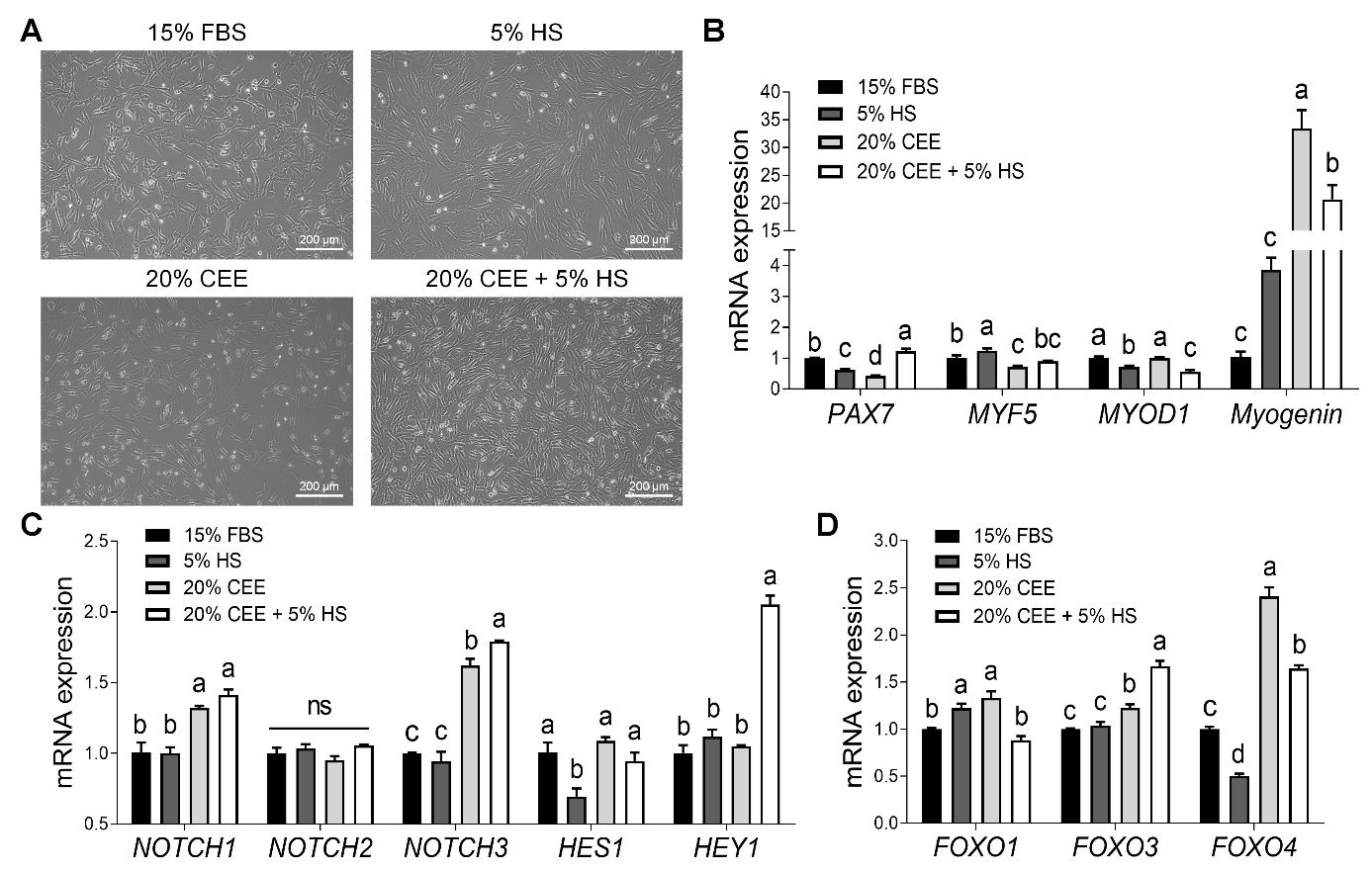


**Fig. S4. CEE distinctly modulates the FOXO/NOTCH signaling axis to sustain self-renewal independent of HS supplementation.** (A) Microscopic images showing the morphology of PMSCs cultured in media containing 15% FBS, 5% HS, 20% CEE, and 20% CEE + 5% HS. Cells maintained in 5% HS alone exhibited more elongated morphology than 15% FBS and 20% CEE + 5% HS. Relative mRNA expression of myogenic regulatory factors (*PAX7*, *MYF5*, *MYOD1*, and *Myogenin*) (B), NOTCH signaling (*NOTCH1*, *NOTCH2*, *NOTCH3*, *HES1*, and *HEY1*) (C), and FOXO signaling related genes (*FOXO1*, *FOXO3*, *FOXO4*) (D). PAX7 and FOXO/NOTCH target genes were upregulated in the CEE + HS group, whereas no comparable increase was observed under HS-only condition. ^a-d^ Different superscripts represent statistically significant differences (*p* < 0.01). All values are represented as the mean ± standard error (SE). CEE, chicken embryo extract; FBS, fetal bovine serum; HS, horse serum.


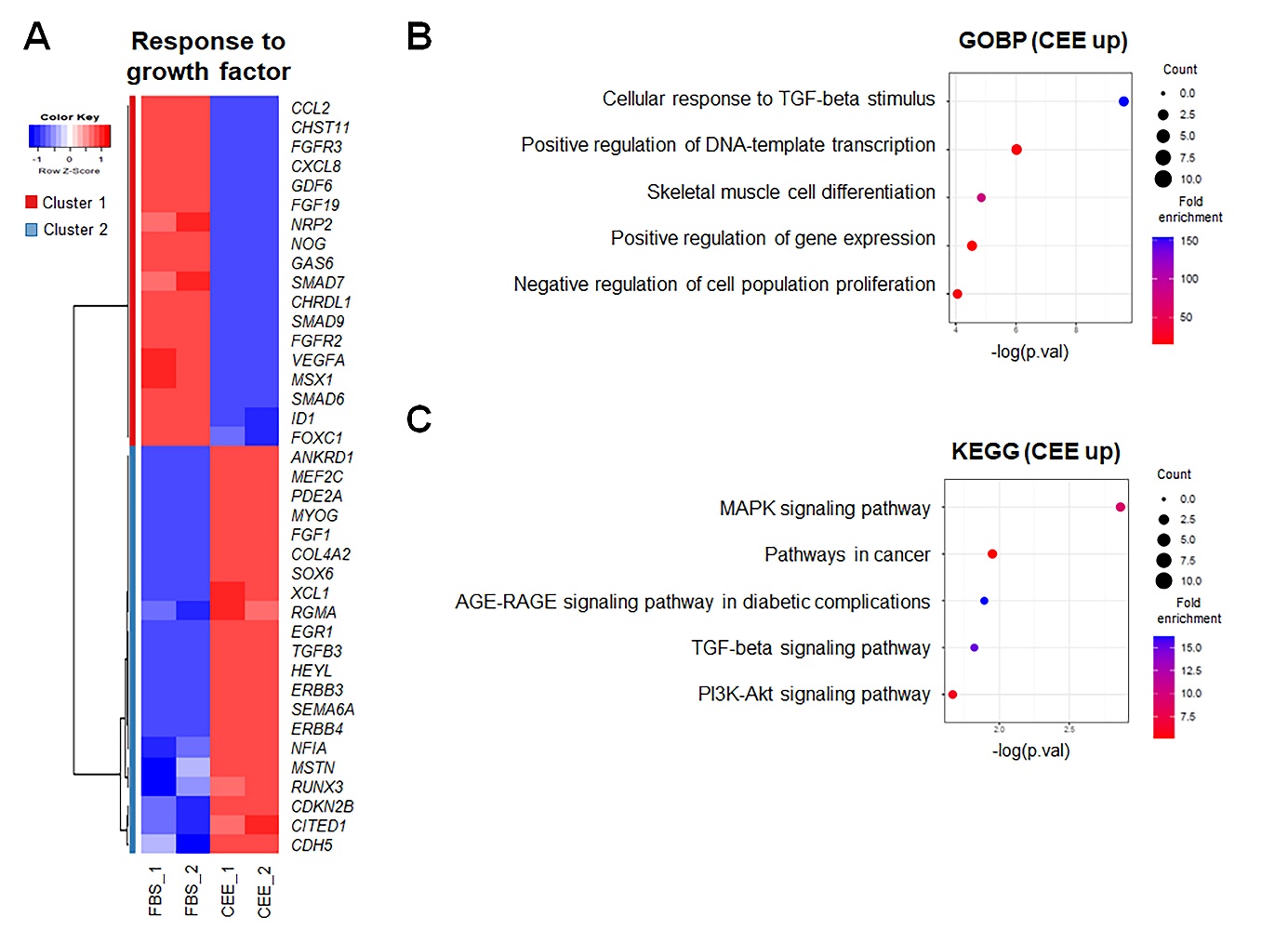


**Fig. S5. Functional enrichment analysis of growth factor related genes in PMSCs in 15% FBS (FBS) and 20% CEE + 5% HS (CEE) media.** (A) Heatmap of DEGs associated with the GO term response to growth factor (GO:0070848). (B and C) GOBP and KEGG pathway enrichment analysis of upregulated response to growth factor genes under 20% CEE + 5% HS groups (CEE) (*n* = 2). DEGs were identified based on FC ≥ 2, *p* < 0.05, and FPKM ≥ 2 and clustered into two groups based on expression profiles. CEE, chicken embryo extract; DEG, differentially expressed gene; FBS, fetal bovine serum; GOBP, Gene Ontology (GO) biological process; HS, horse serum; PMSC, porcine muscle satellite cell.


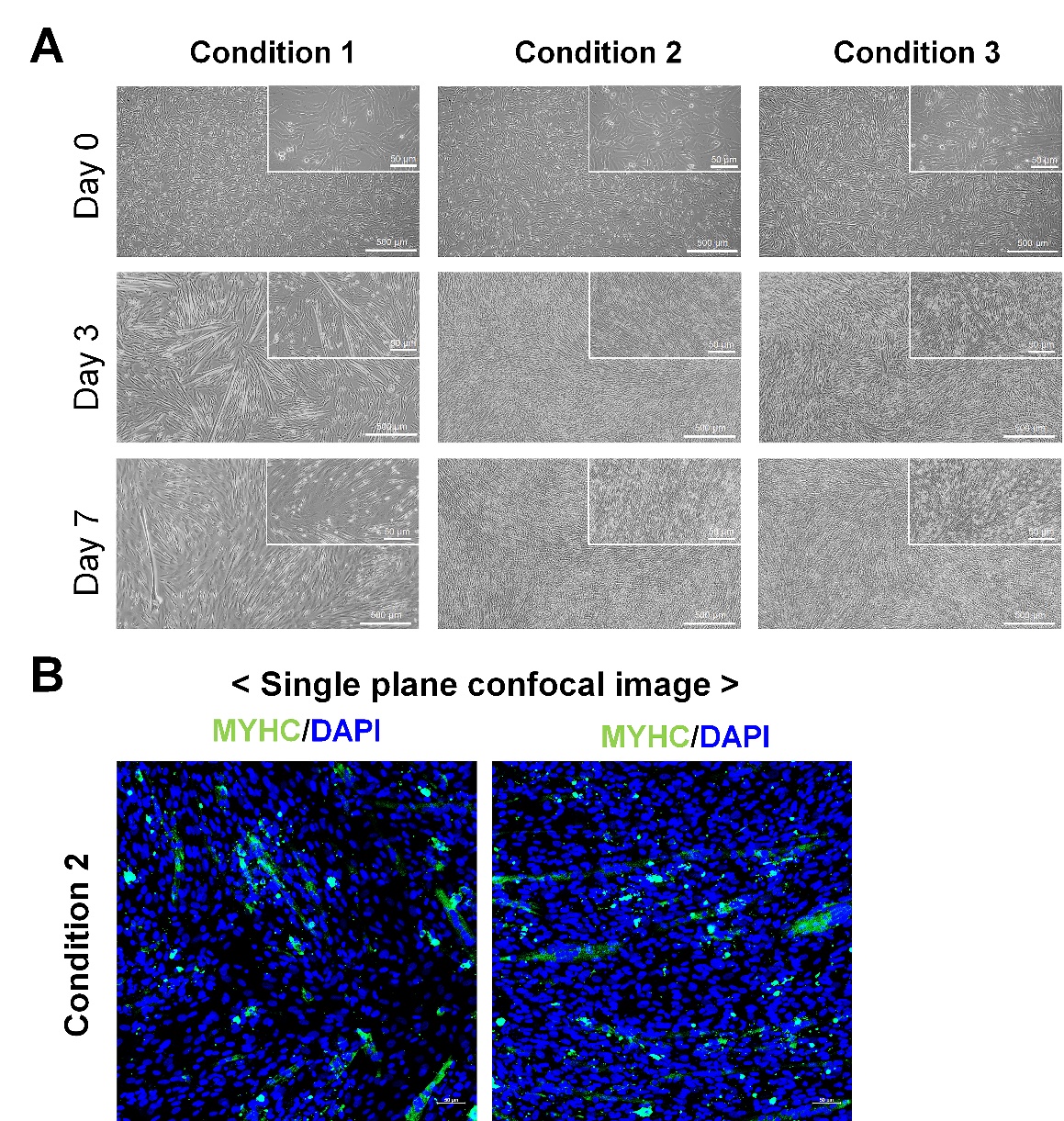


**Fig. S6. The differentiation media composed of 20% CEE + 5% HS formed multilayer structures.** (A) Representative plane confocal images of PMSCs under Condition 2. The immunofluorescent staining of MYHC (green) and DAPI (blue) in PMSCs cultured on Day 3 in Condition 2. (B) The morphological images of progression of PMSCs differentiation under three different conditions on Day 0, 3, and 7. CEE, chicken embryo extract; HS, horse serum; PMSC, porcine muscle satellite cell.

**
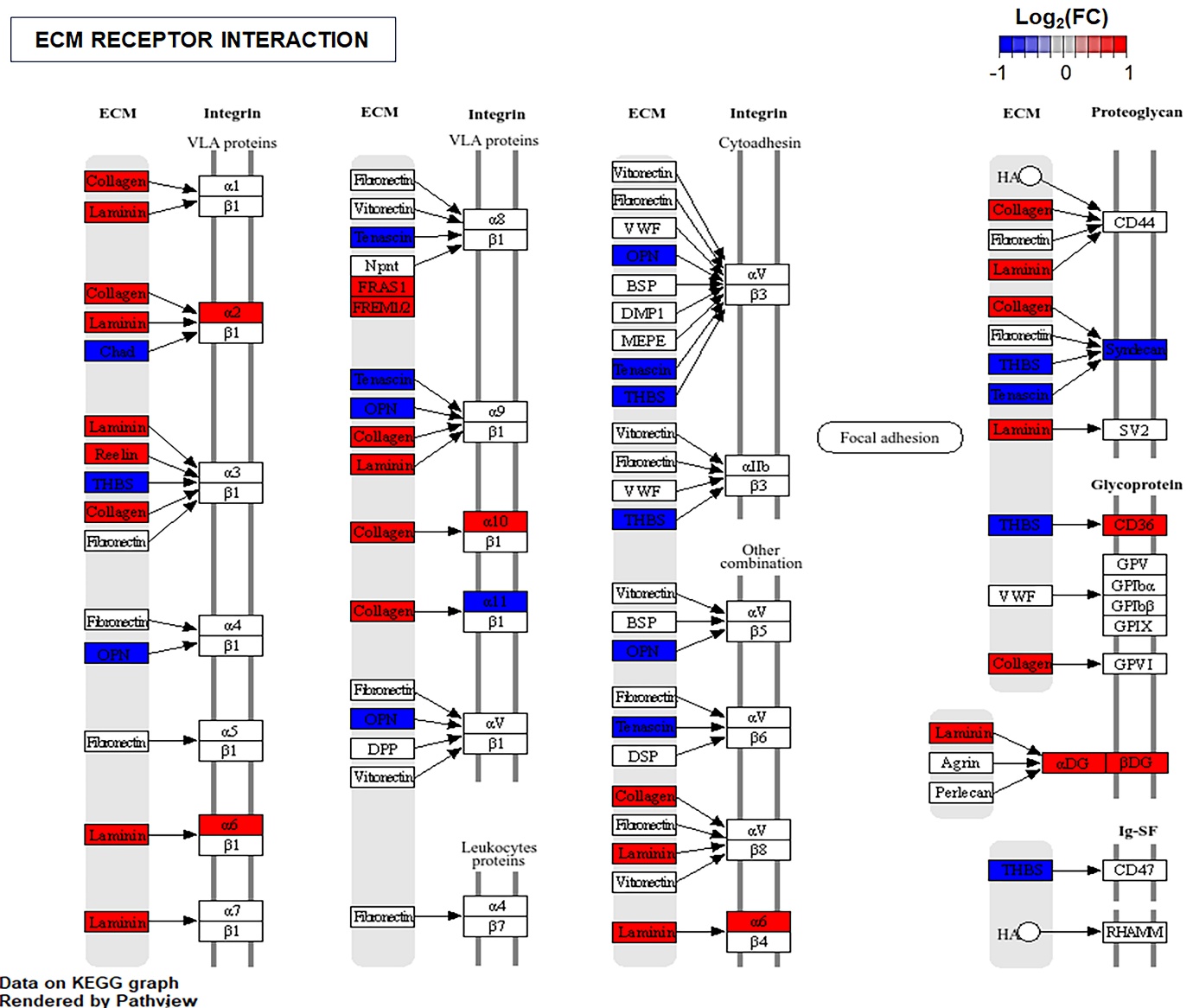
**

**Fig. S7. Visualization of DEGs in the ECM-receptor interaction pathway using KEGG mapping.** DEGs under CEE-based proliferation conditions were mapped to the ECM-receptor interaction pathway using the Pathview in R package. Genes shown in red were upregulated, while those in blue were downregulated relative to the FBS group. The color scale represents log2 fold change (Log_2_FC), with a range of -1 to +1. CEE, chicken embryo extract; DEG, differentially expressed gene; ECM, extracellular matrix.


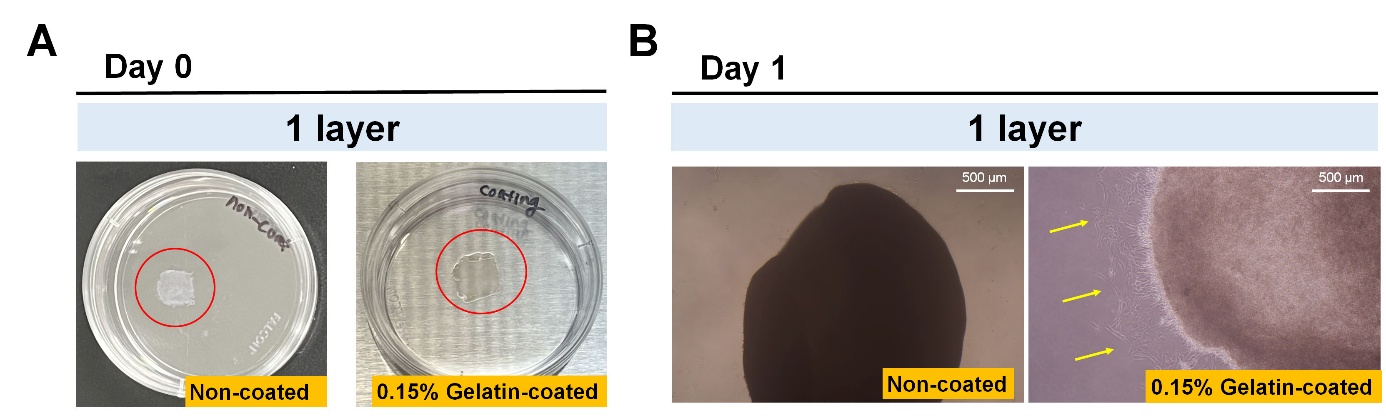


**Fig. S8. Effect of gelatin coating on attachment of transferred cell sheets.** (A) Images of one-layer cell sheets immediately after transfer to 3.5 cm^2^ dishes with or without 0.15% gelatin coating (Day 0). (B) Microscopic images of one-layer cell sheets after Day 1 of additional culture at 3.5 cm^2^ dishes. On the non-coated surface, the cell sheet exhibited aggregation and limited spreading, whereas on the 0.15% gelatin coated surface, cells migrated outward from the sheet edges (yellow arrows) without forming aggregates.

**
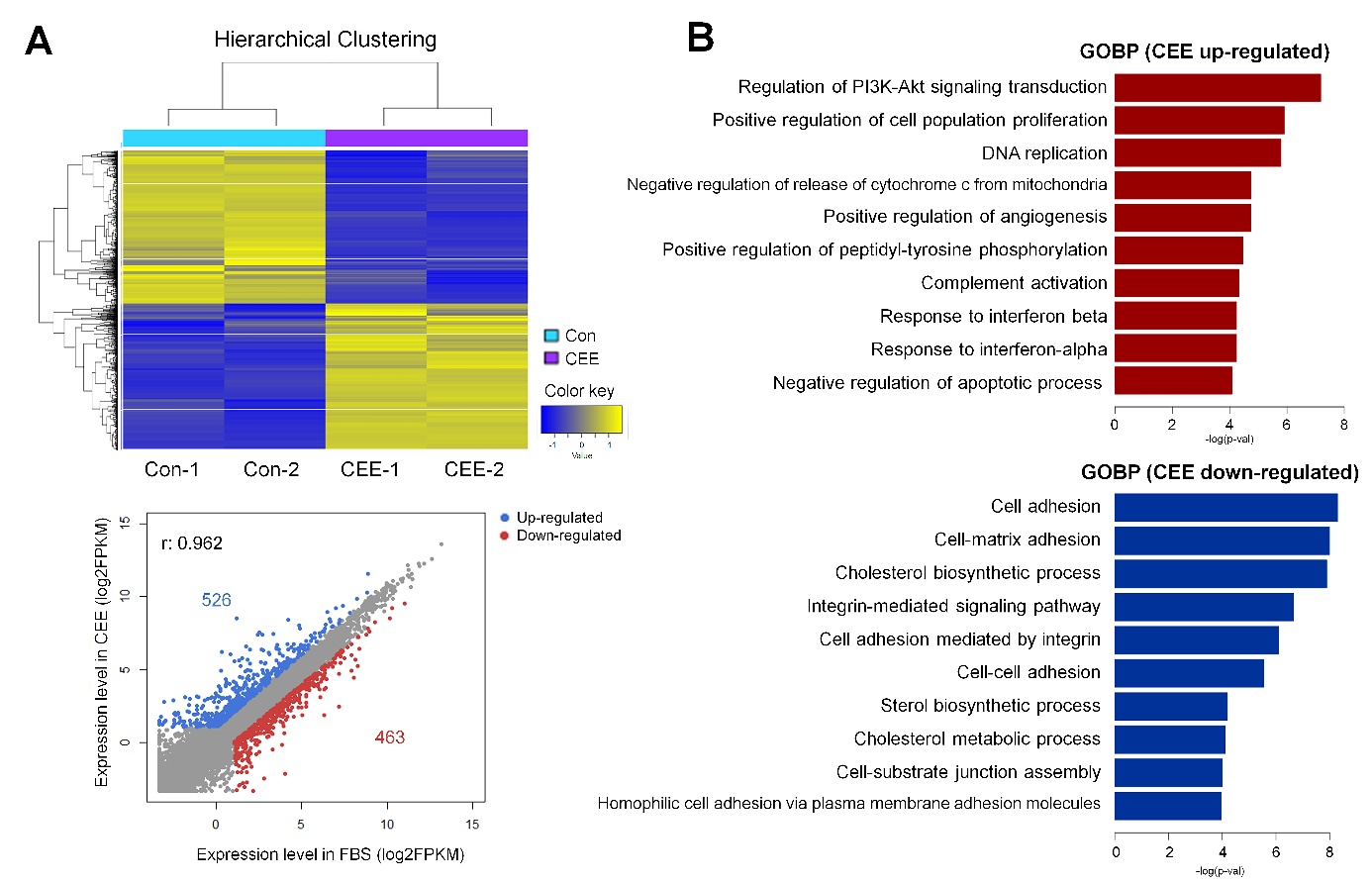
**

**Fig. S9. Functional enrichment analysis of growth factor related genes in PMSCs in control (HS) or 20% CEE + 5% HS (CEE) differentiation media.** (A) Heatmap of DEGs between control (5% HS) and CEE (20% CEE + 5% HS) groups differentiation condition after formation of cell sheets at Day 5. Scatter plot of DEGs indicates significantly upregulated and down-regulated genes in the CEE group. (B) GOBP enrichment analysis of upregulated genes and downregulated genes in the CEE group (*n* = 2). DEGs were identified based on FC ≥ 2, *p* < 0.05, and FPKM ≥ 2 and clustered into two groups based on expression profiles. CEE, chicken embryo extract; DEG, differentially expressed gene; HS, horse serum; PMSC, porcine muscle satellite cell.


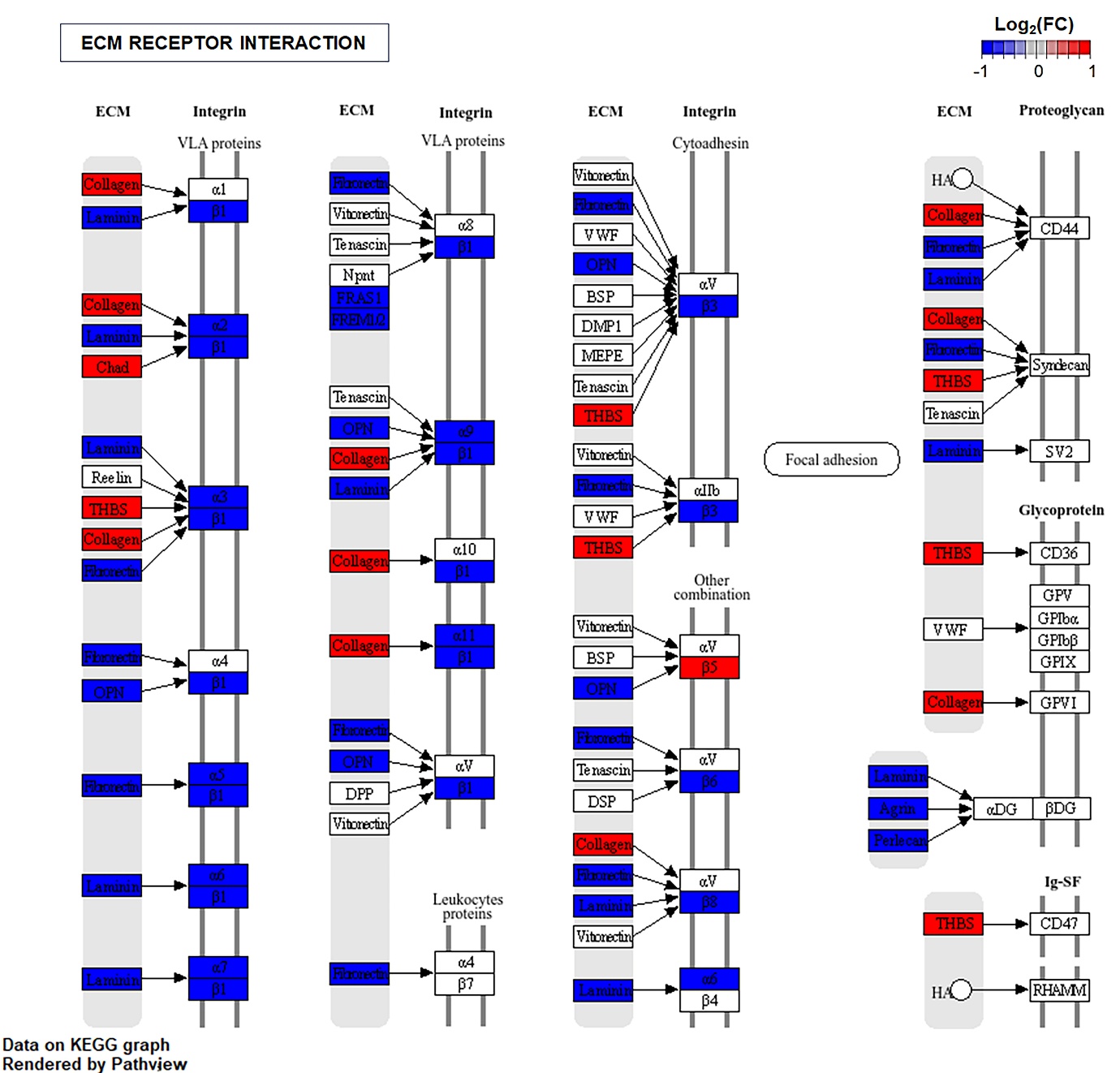


**Fig. S10. Visualization of DEG in the ECM-receptor interaction pathway using KEGG mapping.** DEGs under CEE-based differentiation media (Condition 2) were mapped to the ECM-receptor interaction pathway using the Pathview in R package. Genes shown in red were upregulated, while those in blue were downregulated relative to the FBS group. The color scale represents log2 fold change (Log_2_FC), with a range of -1 to +1. CEE, chicken embryo extract; DEG, differentially expressed gene; ECM, extracellular matrix; FBS, fetal bovine serum; FC, fold change.


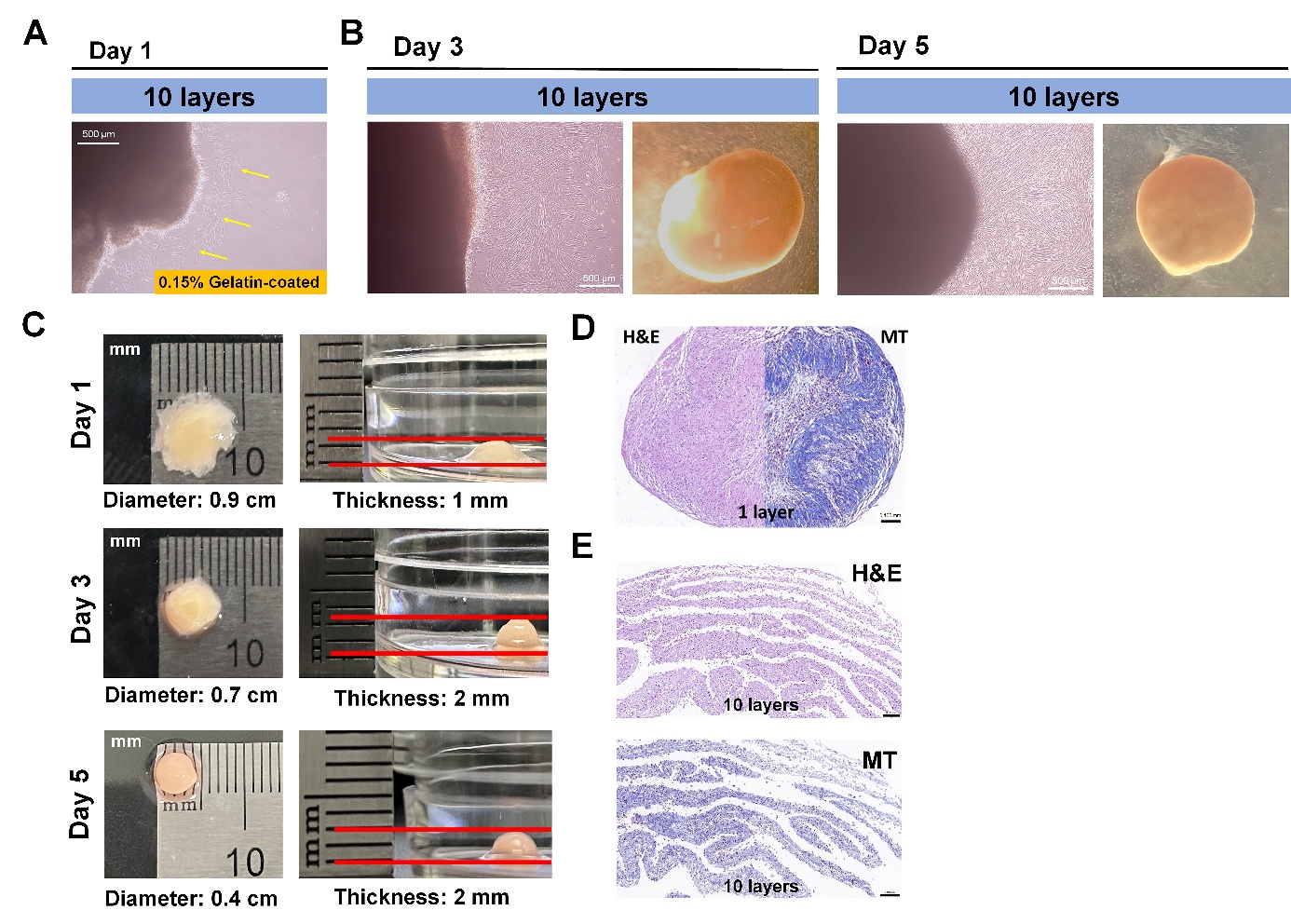


**Fig. S11. Fabrication and characterization of small-scale multilayered cell sheets as a proof-of-concept model for cultured meat.** (A) The 10-layer cell sheet on the gelatin-coated dish showed no aggregation and exhibited continuous outward cell growth from the periphery (yellow arrows) after 1 day. (B) Morphological analysis of 10 layered cell sheet constructs observed on Day 3 and 5 of extended culture in chicken embryo extract differentiation media. (C) Diameter and thickness of laminated cell sheets constructs at Day 1, 3, and 5. (D) Histological analysis of single-layer cell sheet and (E) 10 layered cell sheet construct using hematoxylin and eosin (H&E) staining and Masson’s trichrome (MT) staining. In H&E staining, nuclei are visualized in blue to dark purple, while the cytoplasm and other tissue components appear in pink tone. The MT staining visualized collagen in blue, nuclei in black, and muscle fibers and cytoplasm in red or pink tone.

**SUPPLEMENTARY TABLE**

**Table S1.** Primer sequences for RT qPCR

| Gene name | Primer sequences | Accession number | Length(bp) |
| --- | --- | --- | --- |
| *GAPDH* | F: ACCCAGAAGACTGTGGATGG  R: AAGCAGGGATGATGTTCTGG | NM_001206359.1 | 79 |
| *PAX7* | F: TCCAGCTACTCCGACAGCTT  R: TGCTCAGAATGCTCATCACC | XM_021095458.1 | 100 |
| *MYF5* | F: AGACGCCTCAAGAAGGTCAA  R: AGCCTCTGGTTGGGGTTAGT | NM_001278775.1 | 74 |
| *MYOD* | F: GTGCAAACGCAAGACCACTA  R: GCTGATTCGGGTTGCTAGAC | NM_001002824.1 | 128 |
| *MYOG* | F: CCACTTCTATGACGGGGAAA  R: GGTCCACAGACACGGACTTC | NM_001012406.1 | 203 |
| *SPRY1* | F: AATACACAGAAGGGCCGTCA  R: GCTGGTAGGTCTATGCTCGT | XM_013978914.2 | 131 |
| *NOTCH1* | F: TCCTTTACCTGCCTGTGTCC  R: AGGGCAGGTACACTTGTAGG | XM_021081037.1 | 135 |
| *NOTCH2* | F: ACTACGGGGAGAAGTCAGCT  R: CTTGAACACACTGGCGGTTG | XM_021090691.1 | 129 |
| *NOTCH3* | F: GAACATGGCCAAGGGTGAGA  R: CCTTCAGTCGTTTGGCCTCT | XM_021083631.1 | 86 |
| *HES1* | F: GGAGAGGCGGCAAAGGTATT  R: TGGGGATGAGGAAGGCAAAC | NM_001195231.1 | 76 |
| *HEY1* | F: GTTTGGGGTTTCGGGAATGC  R: CGAAGAGGGTCAGAGGCATC | XM_005663011.3 | 82 |
| *FOXO3* | F: CCTTCAGCAGCACAGTGTTT  R: TGCGACATGGAAGAGAAGGT | NM_001135959.1 | 106 |
| *FOXO4* | F: CGCCAAGCCAAGATCGAATG  R: GAAGTCCAGTCCTTCGCCTC | XM_003135172.4 | 119 |
| *MYHC1* | F: AGGAAAGTCGCAGAACAGGA  R: TCCATCTCTCCCTGGATTTG | NM_001104951.2 | 131 |
| *MYHC2* | F: CCCTGAATGACACAGTGGTG  R: CAGTTTGAGCCCCAGAGAAG | NM_214136.1 | 81 |

**SUPPLEMENTARY VIDEOs**

**Video Legends:** Video 1 and Video 2 present Z-stack confocal images of multilayered MYHC-positive myotubes formed under Condition 2 and Condition 3, respectively.
